# Tunable Sulfated Alginate-based Hydrogel Platform with enhanced anti-inflammatory and antioxidant capacity for promoting burn wound repair

**DOI:** 10.1186/s12951-023-02144-2

**Published:** 2023-10-24

**Authors:** Can Huang, Lanlan Dong, Baohua Zhao, Shurun Huang, Yifei Lu, Xiaorong Zhang, Xiaohong Hu, Yong Huang, Weifeng He, Yong Xu, Wei Qian, Gaoxing Luo

**Affiliations:** 1 Institute of Burn Research, State Key Laboratory of Trauma, Burn and Combined Injury, Chongqing Key Laboratory for Disease Proteomics, Southwest Hospital, Army Medical University, Chongqing, 400038 China; 2 Department of Burns and Plastic Surgery, the 910th Hospital of Joint Logistic Force of Chinese People’s Liberation Army, Quanzhou, 362000 Fujian China; 3 Orthopaedic Institute, Medical College, Soochow University, Suzhou, 215006 P. R. China

**Keywords:** Anti-inflammatory hydrogels, Wound healing, Chemokines, ROS, Macrophages

## Abstract

Amidst progressive advancements in tissue engineering, there has been a significant enhancement in the efficacy of anti-inflammatory hydrogel dressings, addressing a myriad of clinical challenges on wound healing. A frequent complication during the initial stages of deep second-degree burn wound healing is the onset of an inflammatory storm, typically occurring without effective intervention. This event disrupts normal biological healing sequences, leading to undesirable regression. In response, we have customized a tunable, multidimensional anti-inflammatory hydrogel platform based on sulfated alginates (Algs), loaded with Prussian blue (PB) nanozymes. This platform competently eliminates surplus reactive oxygen species (ROS) present in the wound bed. Algs, functioning as a mimic of sulfated glycosaminoglycans (including heparin, heparan sulfate, and chondroitin sulfate) in the extracellular matrices (ECM), demonstrate a high affinity towards inflammatory chemokines such as interleukin-8 (IL-8) and monocyte chemotactic protein-1 (MCP-1). This affinity effectively impedes the infiltration of inflammatory cells into the wound. Concurrently, Algs markedly modulate the macrophage phenotype transition from M1 to M2. Ultimately, our potent anti-inflammatory hydrogels, which strategically target inflammatory chemokines, M1 macrophages, and ROS, successfully attenuate dysregulated hyperinflammation in wound sites. Precise immunomodulation administered to deep second-degree burn wounds in mice has demonstrated promotion of neovascular maturation, granulation tissue formation, collagen deposition, and wound closure. Our biomimetic hydrogels, therefore, represent a significant expansion in the repertoire of anti-inflammatory strategies available for clinical practice.

**Supplementary Information:**

The online version contains supplementary material available at 10.1186/s12951-023-02144-2.

## Introduction

Burn injuries annually impose a substantial economic impact on society [1]. Severe burns, in particular, typically result in suboptimal healing or scarring, which hampers the patient’s reintegration into normal social life [2]. Conservative therapy, such as infection control and wound dressing coverage, are possible for deep second-degree burn wounds as opposed to third-degree burn wounds. However, they continue to represent overwhelming clinical management challenges even in contemporary medicine [3]. The inflammatory storm that occurs during the initial phase of wound healing can exacerbate tissue damage and disrupt natural healing processes, including timely vascular formation and orderly collagen arrangement [4]. Consequently, deep second-degree burn wounds often necessitate surgical intervention, leading to considerable pain and added burden for the patient [4–6]. Given these circumstances, immunomodulatory hydrogels targeting M1 macrophages, ROS, and inflammatory chemokines emerge as potential novel therapeutic modalities for the treatment of deep second-degree burn wounds.

During the inflammatory phase of wound healing, necrotic tissue cells and resident immune cells at the injury site release a range of chemokines [7]. These signaling molecules recruit and activate inflammatory cells, creating a chemotactic gradient that facilitates invasion of blood-derived immune cells, which is critical in physiological healing process to defend against infection and debride damaged tissue [7, 8]. However, in deep second-degree burn wounds, the early stages of healing readily become stalled due to severe tissue damage, resulting in an unchecked inflammatory response [9]. This leads to an overactivation of neutrophils and monocytes/macrophages, causing further tissue destruction and a surge in the production of inflammatory mediators, including chemokines [9, 10]. The persistent chemotactic gradient, primarily derived from IL-8 and MCP-1, aggravates the invasion of these immune cells into the wound bed, perpetuating the vicious cycle of burn wound inflammation [10–12]. Chemokines possess the notable ability to bind to ECM glycosaminoglycans (GAGs) such as heparin or heparan sulfate, a process mediated by electrostatic interactions [13]. In this context, the sulfation degree of GAGs regulates multiple binding events, altering chemokine distribution within the ECM, and consequently governing immune cell activation and migration [13, 14]. Thus, GAG-based biomaterials may therapeutically mitigate the inflammation in deep second-degree burn wounds by modulating the chemokines concentrations in the tissue.

Alginates (Alg) hydrogel dressings, known for their superior biocompatibility, are widely applied in clinical practice due to their exceptional ability to maintain tissue hydration and promote autolytic debridement of necrotic eschar tissue in burn wounds [15, 16]. However, Alg itself exhibits limited anti-inflammatory capacity in vitro. Sulfated alginates (Algs), mimicking ECM GAGs, possess heparin-like properties [17]. And Algs with varying sulfation degrees present distinct sequestration capacities for chemokines, which modulate the inflammatory infiltration in wounds [18–20]. The sulfation degree of Alg, which guides a sequence of binding events to chemokines, holds potential for immunomodulation in deep second-degree burn wounds. Therefore, Algs hydrogel dressings are promising as an effective “molecular sink” to sequester abundant chemokines from inflammatory wound bed, thus hindering the constant recruitment of immune cells and ultimately interfering with the inflammatory process.

Macrophages, as innate immune cells, play crucial functions in wound healing, host defense, and immune regulation [21–23]. And macrophages are highly plastic cells, traditionally classified as M1 macrophages, which vigorously propel inflammatory responses, and M2 macrophages, which robustly strengthen immune modulation and tissue remodeling [24–26]. The polarization of macrophages is largely dependent on the wound microenvironment, which varies throughout the healing process, influencing the phenotype of macrophages and consequently their functions [27]. The persistence of adverse factors in the wound, such as elevated levels of inflammatory cytokines, hyperglycemia, and bacterial infections, will restrict the transformation of pro-inflammatory M1 macrophages into anti-inflammatory M2 macrophages. And the healing process is then stuck in the inflammatory phase, impairing epithelial regeneration, collagen deposition, and neovascularization, which, in turn, precludes its transition to the repair and remodeling phase [24–28]. Therefore, stimulating the polarization of M1 macrophages persistent in deep second-degree burn wounds to M2 macrophages has emerged as a pressing challenge. In this context, we demonstrate that Algs could induce a phenotype shift from M1 to M2, thereby alleviate the overinflamed environment in deep second-degree burn wounds.

In addition to inflammatory chemokines and M1 macrophages, oxidative stress is also deeply involved in inflammation of burn wounds [29, 30]. Three primary sources of reactive oxygen species (ROS) in burn wounds include the respiratory burst of neutrophils, imbalance of xanthine oxidase system, and complement activation [31, 32]. Excess oxygen radicals directly attack cellular lipids, proteins, and DNA, instigating cellular damage and death, which subsequently exacerbates burn wound tissue impairment and spurs a deteriorating inflammatory cascade reaction [33–37]. This aggravated inflammation, in turn, stimulates the overproduction of ROS, provoking a destructive inflammatory cycle and undesirable healing outcomes [38, 39]. Consequently, the elimination of excessive ROS in wound bed becomes essential for deep second-degree burn wounds. Prussian blue, with remarkable detoxification function against radioactive substances, is widely used in clinical practice [40]. Intriguingly, Prussian blue nanoparticles (PBNPs) are also function as antioxidant nanozymes, mimicking the peroxidase (POD), catalase (CAT), and superoxide dismutase (SOD), thereby enabling rapid and efficient scavenging of ROS in burn wounds [41]. Meanwhile, PBNPs can relieve intracellular oxidative stress and enhance the activity of intracellular antioxidant enzymes, such as SOD1 [42]. On top of these advantages, PBNPs are functioning adequately in physiological solutions and have already shown therapeutic effects in acute pancreatitis, ischemic stroke, acute kidney injury, and inflammatory bowel disease [43–47]. Hence, we incorporated PBNPs into Algs hydrogels to further augment their anti-inflammatory activity.

Based on the above considerations, we constructed an innovative anti-inflammatory hydrogel platform. This platform is founded on tunable sulfated alginates (Algs) with notable chemokine sequestration performance, and on Prussian blue (PB) nanozymes renowned for their excellent antioxidant activity. We investigated the effects and underlying mechanisms of this hydrogel platform on deep second-degree burn wounds, aiming to provide new therapeutic modalities for the clinical treatment of these wounds. Our study serves as a valuable experimental reference for further exploration of hydrogel applications in the management of refractory wounds.

**Fig. 1:**
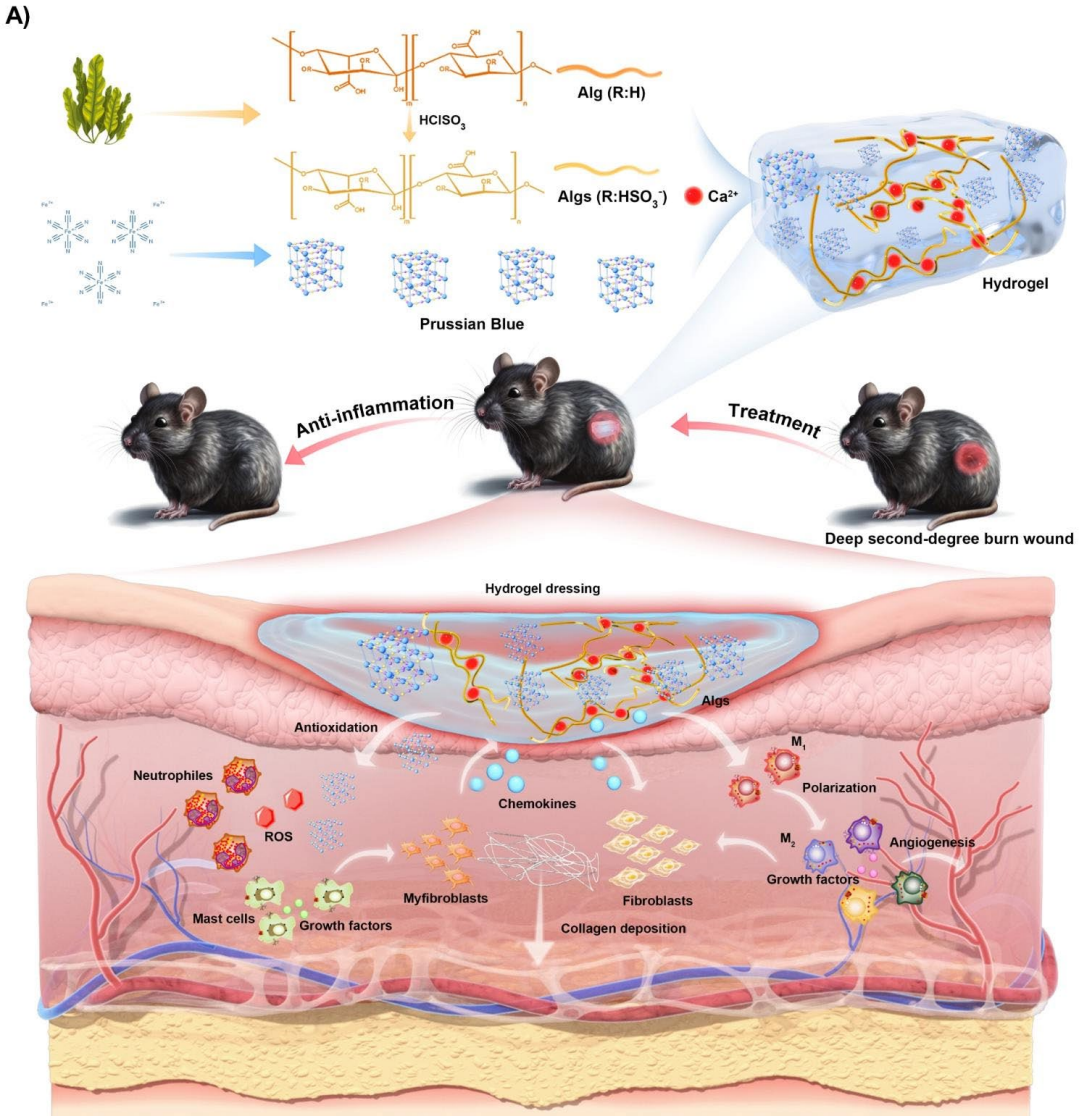
Schematic illustration of the preparation process and therapeutic mechanism of PB@Algs-H hydrogel in deep second-degree burn wound repair in vivo

## Materials and methods

### Synthesis and characterization of Algs

Sulfated alginate (Sigma, USA) was prepared according to the literature [20]. Briefly, 99% HClSO_3_ (Sinopharm Chemical Reagent Co., China) was diluted in formamide (Aladdin, China) at concentrations 1% (Algs-L) and 2% (Algs-H) with a total volume of 40 ml and then added dropwise into 1 g alginate premixed in 20 ml formamide. The reaction was carried out at 60 °C with agitation for 2.5 h. The product was precipitated with cold acetone and centrifuged at 4500 rpm for 10 min followed by dissolution in MilliQ. pH was adjusted to 7.0 by 5 M NaOH during dissolution of sulfated alginate. Samples were purified by dialyzing against 0.1 M NaCl and then MilliQ and finally lyophilized. Sulfation of alginate was confirmed by fourier transform infrared spectroscopy (FTIR, Nicolet-6700, USA). Elemental analysis of sulfur content was taken by inductively coupled plasma optical emission spectrometer (ICP-OES, ThermoFisher ICP-7200, USA).

### Preparation and characterization of PBNPs

PBNPs were prepared as previously described [42]. Typically, 3.0 g polyvinyl pyrrolidone (PVP, Sigma, USA) was dissolved in 0.1 M HCl with a total volume of 40 ml, followed by the addition of 110 mg potassium hexacyanoferrate (III) (K_3_[Fe(CN)_6_], Sigma, USA) with agitation for 1 h. The mixed solution was then placed in a water bath (80 °C) for 20 h. Finally, PBNPs were harvested via centrifugation and repeated wash by MilliQ and ethanol. TEM (JEM-1400, Japan) was conducted to characterize the size and morphology of PBNPs. A dynamic light scattering (DLS) particle size analyzer (Malvern-2000, USA) was utilized to determine the hydrophilic diameter and zeta potential of PBNPs. The UV-vis spectra of PBNPs were acquired with a Hitachi U-3010 spectrometer.

### Fabrication and characterization of the Hydrogels

Different concentrations of calcium chloride (CaCl_2_, Aladdin, China) were added dropwise into the Algs or Alg solution (2 wt%) at a volume ratio: 1:2 under stirring. The hydrogels can be formed within 40 min. The CaCl_2_ concentrations were 50 mM (Alg), 60 mM (Algs-L), 90 mM (Algs-H), and 85 mM (PB@Algs-H), respectively. For the fabrication of PB@Algs-H hydrogels, PBNPs was premixed with CaCl_2_ at a concentration of 200 µg/ml.

Rheological measurements were performed by a TA DHR-2 rheometer. Storage (G’) and loss (G’’) modulus of the hydrogels were monitored with time under 0.5–1% strain and 1 Hz at room temperature. All measurements were conducted within the linear viscoelastic range and repeated at least three times. Scanning electron microscopy (SEM, Crossbeam 340, Germany) was performed to characterize the morphology of the hydrogels.

To investigate the swelling behavior of the hydrogels, the dried hydrogels (W_0_) were immersed in PBS solution at 37 °C for 24 h. After reaching an equilibrium, the samples were weighed again (W_t_). Swelling ratios were calculated by S (%) = (W_t_ − W_0_)/W_0_ × 100.

The kinetic properties of PBNPs release form PB@Algs-H hydrogels were examined with a transwell plate. 600 µl PB@Algs-H hydrogel in transwell inserts was incubated with 1 ml PBS in a shaking bath (100 rpm) at 37 °C. Samples were extracted at specific time intervals within 48 h and replaced with equal fresh medium. The PBNPs concentrations in the medium was measured at 701 nm using a microplate reader.

### In Vitro Biocompatibility of the Hydrogels

HUVECs, NIH 3T3, and Raw264.7 cells were cultured in DMEM (Hyclone, USA) supplemented with 10% fetal bovine serum (FBS, Hyclone, USA) in a 5% CO_2_ atmosphere at 37 °C. Live/Dead staining was executed to evaluate the cell cytotoxicity by co-culturing Raw264.7 with hydrogels after 24 h. Live cells were stained with green fluorescent calcein-AM and dead cells were stained with red fluorescent PI.

CCK-8 was taken to assess the cell proliferation of HUVECs, NIH 3T3, and Raw264.7 co-cultured with different hydrogels. In brief, 1 × 10^4^ cells were seeded in a 96-well plate, and 50 µl hydrogels were placed in culture medium. After 24 h, CCK-8 reagents were added for 1 h. Optical density (OD) was detected at 450 nm and repeated three times.

A hemolysis test was conducted to assess the blood compatibility of the hydrogels. Briefly, 500 µl mouse blood was centrifuged (4 °C, 2000 rpm, 10 min), and the collected erythrocytes were washed three times with PBS and then re-suspended with PBS. Next, 100 µl hydrogel samples were added into 900 µl re-suspended erythrocytes in a tube at 37 °C for 4 h. A positive control (H_2_O) and a negative control (PBS) were also included in the experiment. Finally, all samples were centrifuged (2000 rpm, 10 min) and the supernatant was taken to measure the absorbance at 545 nm by a microplate reader.

### Multiple enzyme-like activities of PBNPs

The H_2_O_2_ depletion capacity of PBNPs (0, 500, 750, and 1000 ng mL^− 1^) was investigated by a hydrogen peroxide assay kit (Solarbio, China) according to the manufacturer’s instructions. The ability of PBNPs (0, 10, 20, and 40 µg mL^− 1^) for depleting OH· generated by the Fe^2+^/H_2_O_2_ system was assessed by EPR (Bruker-A300, Germany) and DMPO (Sigma, USA) was used as the spin probe. The ability of PBNPs (0, 10, 20, and 40 µg mL^− 1^) for quenching O2^**·−**^ produced by the xanthine/XO system was detected by EPR utilizing DMPO as the O2^**·−**^ trapping agent.

PBNPs can inhibit the generation of fluorescent 2-hydroxyterephthalic acid (TPA-OH), and a TPA-Na/TPA-OH system (MedChemExpress, USA) was used to evaluate the CAT-mimetic activity of PBNPs. Briefly, TPA-Na (10 × 10^− 3^ m) was added into H_2_O_2_ (5 × 10^− 3^ M) with or without PBNPs. The mixture was shaken for 10 min at 45 °C in the dark. Then, the sample was withdrawn and measured by a fluorescence spectrometer (425 nm).

The POD-like activity of PBNPs was investigated by a POD assay kit (Solarbio, China) according to the manufacturer’s instructions. Typically, POD can catalyze the oxidation of guaiacol to produce a tea-brown substance in the presence of H_2_O_2_, which has a maximum absorption at 470 nm. PBNPs was added into the H_2_O_2_/guaiacol system and the absorbance samples was measured by a microplate reader.

The SOD-mimetic activity of PBNPs was evaluated by formazan formation utilizing a SOD assay kit (Nanjing Jiancheng Bioengineering Institute, China). In brief, O2^**·−**^ was generated through xanthine/XO system, which can convert the WST-1 into WST-1 formazan with an absorption perk at 450 nm. The WST-1 formazan concentration was then determined at 450 nm by a microplate reader.

### Intracellular ROS measurements

The ROS-sensitive DCFH-DA (MedChemExpress, USA) was used to detect ROS levels in HaCaT cells. Briefly, 5 × 10^5^ HaCaT cells were pretreated in 6-well plates for 12 h with PBNPs (0, 100, 150, or 200 µg/mL) followed by treatment with 700 µM H_2_O_2_. Untreated cells served as controls. After 6 h of treatment, cells were harvested and stained for 30 min with 5 µM DCFH-DA (MedChemExpress, USA). The intracellular ROS levels in HaCaT cells were then quantitatively analyzed by flow cytometry (Applied Biosystems, USA).

### In Vitro Macrophage phenotype modulation of the Hydrogels

Bone marrow derived macrophages (BMDMs) were obtained from the femur and tibia in C57 mice. The purity of BMDMs was confirmed with anti-CD11b (Biolegend, USA) and anti-F4/80 (Biolegend, USA) antibodies flow cytometry (Applied Biosystems, USA). To induce M1 polarization, the BMDMs were stimulated with 100 ng ml^− 1^ lipopolysaccharide (LPS, Sigma, USA) and 10 ng mL^− 1^ IFN-γ (PeproTech, USA) for 24 h. The M1 polarization of BMDMs was identified with anti-CD86 (Biolegend, USA) and anti-F4/80 antibodies by flow cytometry.

Next, to investigate the effect of the hydrogels on M1 macrophages polarization, cells were co-cultured with different hydrogels for 48 h, respectively. The harvested cells were used for subsequent experiments. Cells were stained with anti-CD206 antibodies (Biolegend, USA) and anti-F4/80 antibodies to quantitatively analyze the proportion of M2 macrophages by flow cytometry. Data were analyzed by FlowJo software.

Immunofluorescent staining was conducted to analyze the expression of M2 gene markers using rabbit anti-CD206 (CST, USA) and rabbit anti-Arg-1 (GeneTex, USA) primary antibodies followed by incubation with goat anti-rabbit-Alexa Fluor-647 (CST, USA) and goat anti-rabbit-Alexa Fluor-488 (CST, USA) secondary antibodies. Fluorescence imaging was implemented with a CLSM (Zeiss, Germany).

Relative mRNA expression of M1 macrophage marker genes (iNos,TNF-α, IL-6, and Il-1β) and M2 macrophages marker genes (Arg1, Il-10, and VEGF) was quantified by RT-qPCR (Bio-rad, USA), and normalized to expression of ribosomal protein L13a (Rpl13a).

Finally, the expression of M1 macrophages markers (iNOS and MMP-9) and M2 macrophages markers (CD206 and Arg-1) were detected by western blot. The primary antibodies in this experiment were as follows: rabbit anti-β-Actin antibody (CST, USA), rabbit anti-CD206 antibody (CST, USA), rabbit anti-Arg-1 antibody (CST, USA), rabbit anti-iNOS antibody (CST, USA), and rabbit anti-MMP-9 antibody (CST, USA).

### In vivo effects of the hydrogels on Deep Second-Degree burn wounds

Eight-week-old male C57 mice were purchased from the Animal Center of the Army Medical University. All animal experimental procedures were approved by the Laboratory Animal Welfare and Ethics Committee of Army Medical University. To acquire accurate burn area and depth, an electric scalding instrument (Jinan Yiyan Technology Development Co., China) with controlled temperature and pressure was utilized for modeling. Firstly, the dorsal hairs of mice were removed. Then, a 1.5-cm diameter iron bar with constant temperature 80 °C and 0.5 kg pressure was placed on the back of the mice skin for 8 s to form a deep second-degree burn wound. The burn area is approximately 5% of the total body surface area (TBSA). Finally, Hematoxylin and eosin staining (H&E) staining was performed to determine the burn depth of mice. Afterwards, the burned mice were randomly divided into five groups (n ≥ 3): control (saline dressing), Alg, Algs-L, Algs-H, and PB@Algs-H group. Wound dressings were replaced at two-day intervals. On days 1, 7, 14, and 21, the wounds were photographed and wound areas were calculated by Image J.

On day 2 after treatment, immunofluorescence staining of IL-8/CXCL15 and MCP-1 (Abcam, USA) were performed to evaluat the adsorption effect of different hydrogels. On day 4, 200 mg wounds tissues were collected to determine the concentrations of IL-6 and TNF-α by enzyme-linked immunosorbent assay (ELISA, Abcam, USA). On day 7, ROS levels in wounds tissue was detected by a ROS assay kit (MedChemExpress, USA). In brief, frozen 8-µm-thick tissue sections were rinsed in PBS and stained for 30 min with 20 µM dihydroethidium (DHE).

On day 7, the wound tissues were harvested and digested with Liberase™ TL (Sigma, USA) to yield a single cell suspension. Cells were then stained with anti-CD206 antibodies (Biolegend, USA) and anti-CD11b (Biolegend, USA) antibodies to quantitatively analyze the proportion of M2 macrophages by flow cytometry. Data were analyzed by FlowJo software. Meanwhile, immunofluorescence staining of iNOS and Arg-1 (GeneTex, USA) were performed to assess the polarization of macrophages.

On day 14, immunofluorescence staining of CD31 (Abcam, USA) were implemented to investigate the presence of neovascularization in the granulation tissue. On day 21, the wound tissues were collected and then fixed in 4% formaldehyde for paraffin embedding. Tissue Sect. (10 μm) were used for histological analysis. H&E staining was conducted to measure the thickness of granulation tissue. Masson’s trichrome staining was carried out to quantify the formation of new collagen.

### Transcriptome analysis

Deep second-degree burned mice were randomly divided into 2 groups: the control group and the PB@Algs-H group (n = 3). On day 7 after treatment, total RNA was extracted from wound tissues by a RNeasy Fibrous Tissue Mini Kit. RNA concentration and quality were determined utilizing the NanodropND-1000 spectrophotometer (Gene Company, USA) and 2100 Bioanalyser (Agilent, USA), respectively. Pure RNA samples were used as starting material for library preparation and sequenced by Illumina HiSeq X10 (Illumina, USA).

Transcripts per million reads (TPM) were employed to calculate the differentially expressed genes (DEGs) between the PB@Algs-H and control groups. Differential expression analysis was performed using DESeq2/DEGseq/edgeR/Limma/NOIseq. Criteria for differential genes were padjust ≤ 0.05. Gene Ontology (GO) (https://github.com/tanghaibao/Goatools) were used for functional enrichment. The target gene set heatmap, Gene Set Enrichment Analysis (GSEA), and protein-protein interaction (PPI) network were calculated to search for critical genes on the website (https://vip.majorbio.com/), and the enrichment score (ES) obtained from GSEA analysis reflected the over-representation of a certain gene set at the top or bottom of a ranked list of genes in the expression dataset of these two groups.

### Statistical analysis

Results were expressed as mean ± standard deviation (SD) from at least 3 independent experiments. Two-tailed Student’s t test or one-way ANOVA (GraphPad Prism 9.0) was used for statistical analysis. The significance of differences was treated as follows: ns, not statistically significant; * represents p < 0.05, ** represents p < 0.01, *** represents p < 0.001, and **** represents p < 0.0001.

## Results and discussion

### Preparation and characterization of Algs and PB Nanozymes

**Fig. 2:**
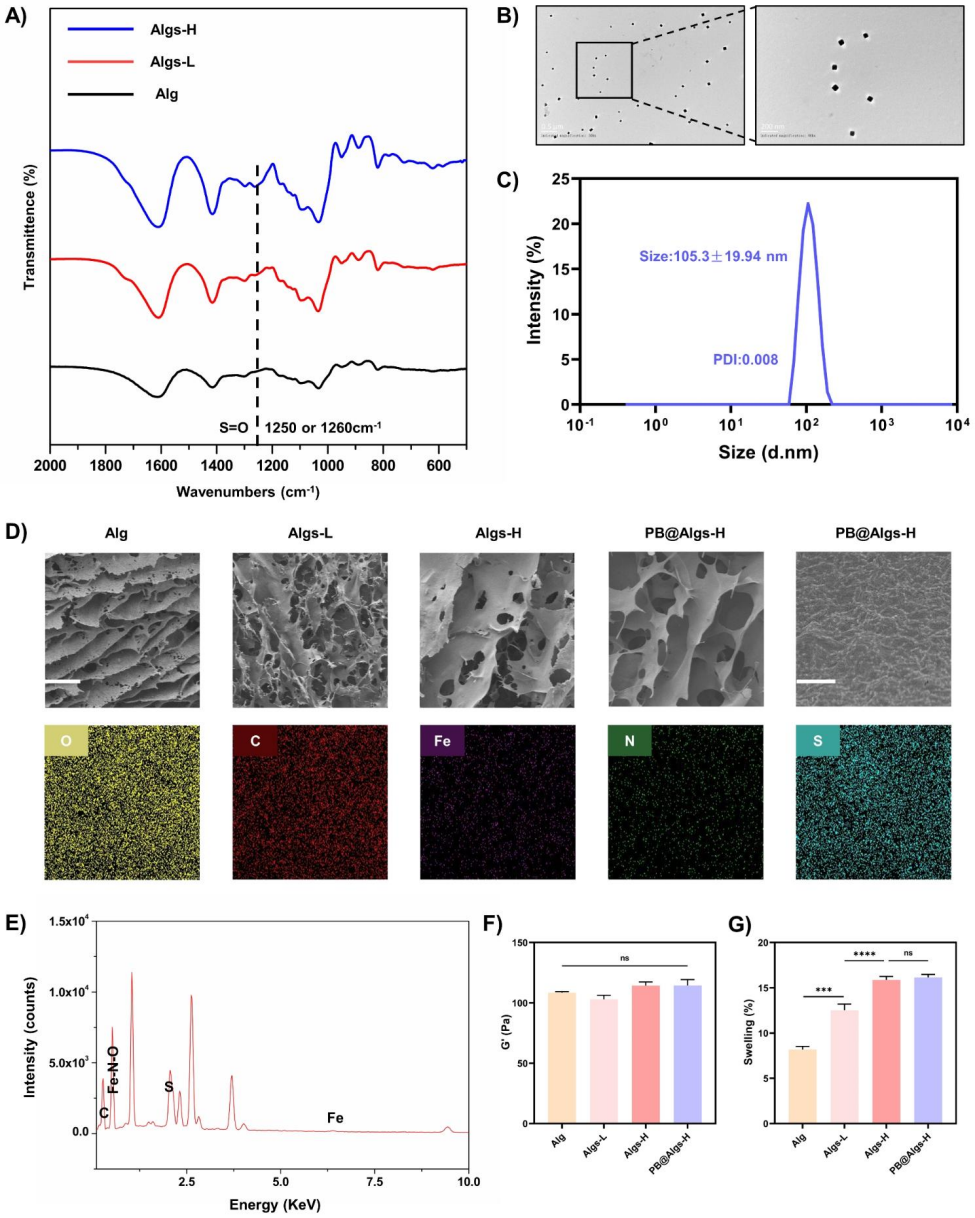
Characterization of hydrogels. (**A**) FTIR spectra of Alg, Algs-L, and Algs-H. Line indicates peaks characteristic to S = O stretching at 1250 cm^− 1^ or 1260 cm^− 1^. (**B**) TEM images of PBNPs. (**C**) The hydrodynamic particle distribution of PBNPs in aqueous media. (**D**) SEM images of hydrogels and element mappings of O, S, Fe, C, and N elements of PB@Algs-H hydrogels. Scale bar: 100 μm and 1 μm. (**E**) The energy-dispersive X-ray spectrum (EDS) of PB@Algs-H hydrogels. (**F**) Storage modulus (G’) of hydrogels. (**G**) Percent mass swelling of hydrogels after 24 h. (n = 3; mean ± s.d.; ns, not statistically significant; ***p < 0.001, ****p < 0.0001)

The Fig. 1 showed a straightforward schematic of the preparation process of Algs and PB nanozymes. Briefly, Algs was synthesized according to the previously protocol in the literature [20]. Algs with different sulfation degrees (Algs-L and Algs-H) were obtained by varying the concentration of chlorosulfonic acid (HClSO_3_) as a contrast to the unsulfated Alg. FTIR spectra of Algs-L and Algs-H at 1250 cm^− 1^ or 1260 cm^− 1^ confirmed the S = O stretching characteristic peak (Fig. 2A). Furthermore, we quantitatively analyzed the sulfur content in Algs-L and Algs-H by elemental analysis (Figure S1D), reflecting the difference in sulfation degree between these two products.

We fabricated the stable PBNPs by a simple hydrothermal method in the literature [42]. The UV−vis spectroscopy of PBNPs revealed a strong absorbance peak at ~ 700 nm attributable to the intermetallic charge transfer from Fe(II) to Fe(III) (Figure S1C). Transmission electron microscopy (TEM) images displayed the well-formed homogeneous square PBNPs, which possessed high monodispersity (Fig. 2B) with average diameter of 54.60 ± 11.96 nm (Figure S2A). And the average hydrodynamic particle diameter of PBNPs was about 105.3 nm with a narrow size distribution (polydispersity index: 0.008) (Fig. 2C), and the zeta potential was about − 14.7 mV (Figure S1B), indicating that PBNPs were well dispersed in aqueous media. Good dispersibility of PBNPs is an overriding prerequisite for their antioxidant activity.

### Preparation, characterization, and Biocompatibility of Hydrogels

**Fig. 3:**
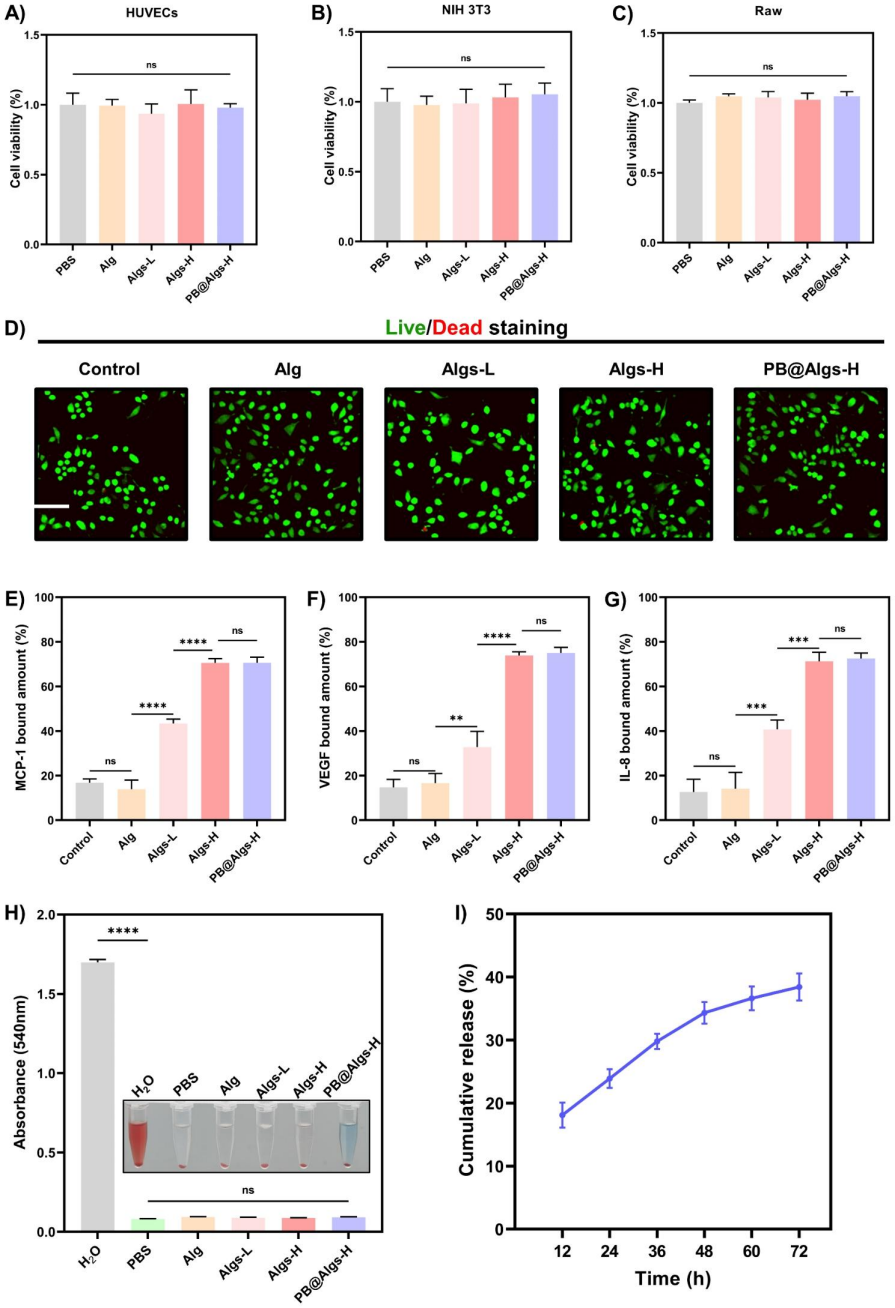
Biocompatibility and chemokine sequestration of hydrogels. **A, B, C**) CCK-8 assay of HUVECs, NIH 3T3, and RAW264.7 cells co-cultured with PBS or hydrogels after 24 h (n = 3). **D**) Confocal microscopy images of live/dead staining assay of Raw264.7 cells co-cultured with PBS or hydrogels after 24 h. Scale bar: 200 μm. **E, F, G**) MCP-1, VEGF, and IL-8 bound amount to hydrogels. One hundred nanograms of MCP-1, VEGF, and IL-8 was added to 50 µl different hydrogels and sequestration was determined after co-incubation with hydrogels for 24 h. **H**) Hemolysis photographs and ratio (%) after exposure to hydrogels. **I**) cumulative release of PBNPs from PB@Algs-H hydrogels. (n = 3; mean ± s.d.; ns, not statistically significant; **p < 0.01, ***p < 0.001, ****p < 0.0001)

Numerous studies have unveiled that one of the reasons for the widespread clinical applications of hydrogels is that their soft, porous nature is in line with that of ECM [48–51], allowing for hydrogel-skin mutual adaptation. Under such circumstances, cells in burn wounds readily migrate and proliferate in response to biological cues without external interference [50–52]. To achieve a stable hydrogel framework while preserving the softness of hydrogels, the concentration of Alg or Algs was maintained at 2%. Meanwhile, the stiffness of hydrogels was uniformed by adjusting the crosslinking agent concentration (Fig. 2F and Figure S2A-E) for excluding their influence on experiment results. And other rheological properties of hydrogels were systematically examined (Figure S2G-I). Ca^2+^ is non-toxic to cells compared to other crosslinking ions, such as Pb^2+^, Cd^2+^, Ba^2+^, etc., and is indispensable for physiological activities of the body. Accordingly, Ca^2+^ was chosen as the crosslinking agent, and the Ca^2+^ concentrations were 50 mM (Alg), 60 mM (Algs-L), 90 mM (Algs-H), and 85 mM (PB@Algs-H), respectively. Among them, the concentration of PBNPs in the PB@Algs-H group was 200 µg/ml, and since the ferric ions acted as a partial crosslinker role, the Ca^2+^ concentration in the PB@Algs-H group was slightly lower than that in the Algs-H group. The introduction of sulfate group imparted more negative charge to Alg and raised the hydrophilicity of Algs hydrogels. As a consequence, the mass swelling ratio of Algs hydrogels was elevated with the increase of sulfate group. And Algs-H hydrogels presented the highest swelling capacity of about 16%, while the PB@Algs-H and Algs-H groups showed no significant difference in swelling behavior (Fig. 2G). The swelling ability of hydrogels endows them with absorbing exudate from the wound bed and fostering the evacuation of necrotic tissue. Scanning electron microscopy (SEM) images revealed that hydrogels in all groups displayed a porous network structure resembling ECM, and element mapping and EDS confirmed that the doped PBNPs were uniformly distributed throughout the Algs-H polymer network (Fig. 2D and E). The steady release of PBNPs from the hydrogel system is an important precondition for their antioxidant effects. We examined the kinetic properties of in vitro release of PB@Algs-H hydrogels (Fig. 3I). At 37 °C, PB@Algs-H hydrogels manifested a sustained and controlled release behavior, reaching a cumulative release of approximately 36% after 72 h of incubation. Therefore, PBNPs will receive gradual release from PB@Algs-H hydrogels and continuously exert antioxidant effects after being deployed on burn wounds.

For practical applications of hydrogel dressings, exceptional biocompatibility is a fundamental requirement [49–51]. To assess the biocompatibility of hydrogels, we substantiated the in vitro safety of hydrogels by the Cell Counting Kit (CCK-8), live/dead staining, and a hemolysis assay. Firstly, mouse-derived macrophages (Raw264.7) were co-cultured with different hydrogels, and live/dead staining assay was executed after 24 h of co-incubation (Fig. 3B). The overwhelming majority of macrophages were alive (green fluorescence) and only few dead cells were observed (red fluorescence) in the control, Alg, Algs-L, Algs-H, and PB@Algs-H groups, indicating that the hydrogels could support cell adhesion and growth. The next CCK-8 experiment indicated that cell viability remained consistency with the control group when HUVECs, NIH 3T3, and Raw264.7 cells were co-cultured with hydrogels for 24 h, suggesting that the hydrogels were free of cytotoxic effects (Fig. 3A-C). Finally, given the inevitable contact of hydrogels with blood, a hemolysis test was taken to further evaluate the hemocompatibility of hydrogels. After incubation with mouse blood, the hydrogel groups did not cause any significant hemolysis compared to the PBS group, while the H_2_O group experienced obvious hemolysis (Fig. 3H).

Collectively, these results illustrated that customized hydrogels presented ECM-mimetic mesh structure with favorable swelling ability and unparalleled biocompatibility, which are ideal candidates for covering, dressing replacement, and debridement of deep second degree burn wounds.

### Anti-inflammatory effects of Hydrogels in Vitro

Chemokines, ROS, and M1 macrophages are considered as major contributors of excessive wound inflammation [12, 24, 30]. We designed an anti-inflammatory hydrogel inspired by biomolecular interactions to ameliorate the abnormal inflammatory environment in deep second-degree burn wounds, thereby resolving a series of clinical challenges regarding the treatment of inordinate inflammatory infiltration in wounds.

### Algs Hydrogels Sequester chemokines

Many growth factors and chemokines, such as vascular endothelial growth factor (VEGF), IL-8, and MCP-1, etc. are termed heparin-binding proteins [17]. And their distribution in tissues is dominated by their complexation with sulfated GAGs of ECM or cell surface proteoglycans. Additionally, the binding affinity was found to depend on the degree of polysaccharide sulfation [17–19]. As such, we provided an approach to manage chemokine levels in tissue to suppress immoderate wound inflammation based on the interaction of GAGs with heparin-binding proteins. Three polysaccharides with different sulfation degrees: Alg (0%), Algs-L (1%), and Algs-H (2%), were synthesized by tuning the concentration of HClSO_3_. Algs hydrogels were examined for heparin-binding proteins capture by co-incubating with IL-8, MCP-1, and VEGF for 24 h (Fig. 3E-G). For IL-8, MCP-1, and VEGF, the Algs-H group adsorbed 71.3%, 70.5%, and 73.9%, respectively, and there was no pronounced difference in the adsorption of the above three proteins between the PB@Algs-H and Algs-H groups, suggesting that the introduction of PBNPs did not affect the capture properties of Algs-H. The Algs-L group scavenged 40.8%, 43.4%, and 32.8% of IL-8, MCP-1, and VEGF correspondingly, which was distinctly lower than the PB@Algs-H and Algs-H groups, and the results should be attributed to the variation in the number of sulfated groups on Alg. While the Alg and control groups appeared to have a certain amount of adsorption of these cytokines, there was no difference between these two groups, indicating that the final results were furnished by in vitro proteins degradation during incubation and there was no actual scavenging in the sense.

IL-8, a neutrophil chemokine, and MCP-1, a monocyte chemokine, are key mediators of inflammation and are associated with the long-term presence of immune cells in burn wound tissue. In contrast, VEGF takes a vital role in the reconstruction of damaged tissues, and even determines the outcome of wound repair. One of the reasons why the clinically favored hydrogel dressings that cover fractured skin and regulate moisture are less effective in deep second-degree burn wounds may be that amplified protease levels in wound tissue deplete pro-regenerative growth factors and accelerate fibroblast senescence [14]. Nevertheless, our established hydrogel platform competently sequesters inflammatory chemokines and thus blocks the aggregation of inflammatory cells to the burn wound bed while shielding the growth factors from protease degradation, which is undoubtedly a great benefit to wound repair.

### PB Nanozymes Scavenge ROS and alleviate intracellular oxidative stress

**Fig. 4:**
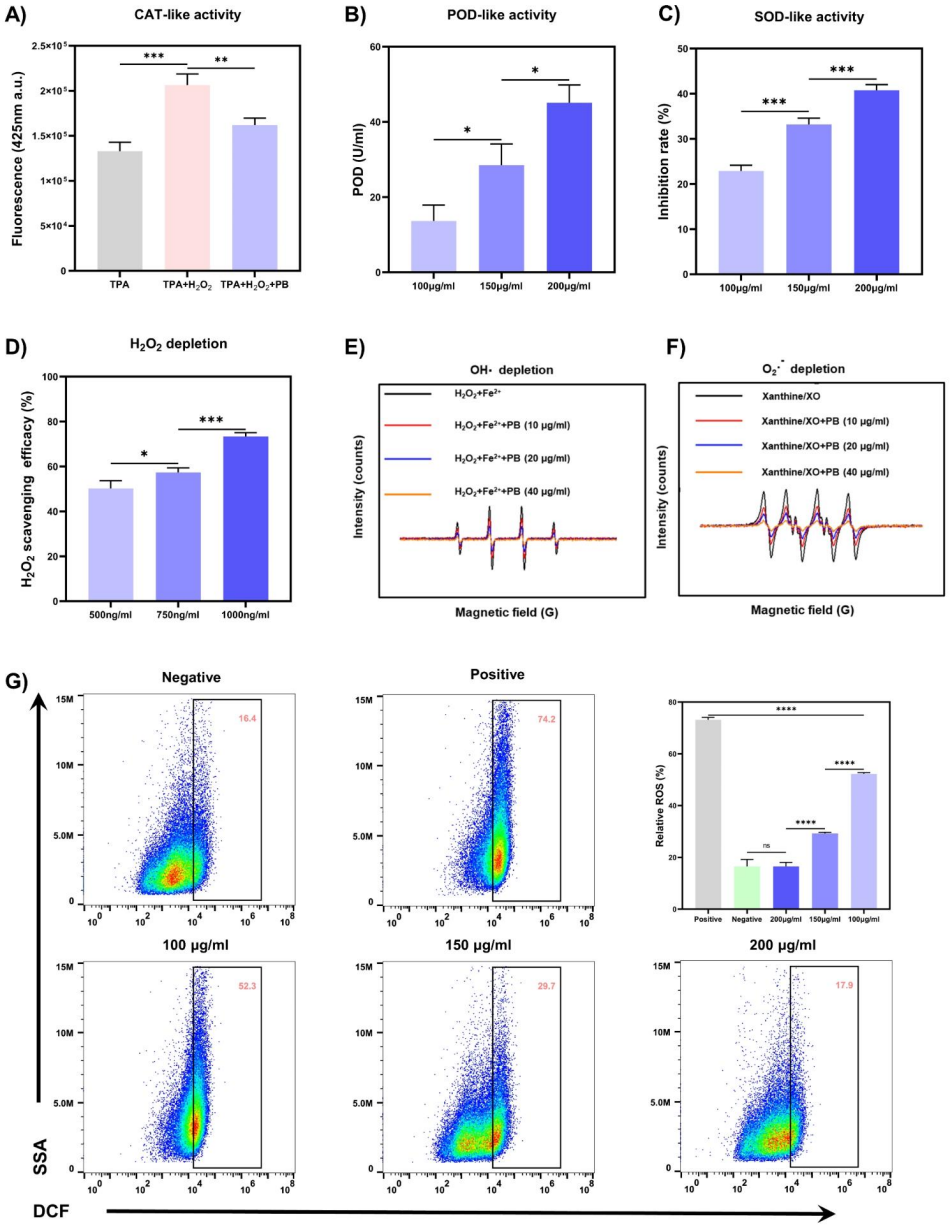
Multiple enzyme-like activities and cytoprotection against ROS of PBNPs. (**A**) CAT-like activity of PBNPs determined by TPA-Na. (**B**) POD-like activity of PBNPs utilizing guaiacol as the chromogenic substrate. (**C**) SOD-like activity of PBNPs determined by an SOD assay kit. (**D**) H_2_O_2_ depletion of PBNPs determined by titanium sulfate method. (**E**) OH· depletion capacity of PBNPs determined by EPR. (**F**) O_2_^**·−**^ depletion of PBNPs determined by EPR. (**G**) ROS in HaCaT cells was detected by flow cytometry combined with DCFH/DCF ROS detecting system. (n = 3; mean ± s.d.; ns, not statistically significant; *p < 0.05, **p < 0.01, ***p < 0.001, ****p < 0.0001)

ROS have been recognized as pivotal mediators in the initiation and progression of wound inflammation [29]. PB nanozymes afford cells protection against ROS-induced oxidative stress by scavenging various ROS including O_2_^**·−**^, OH**·**, and H_2_O_2_ [37]. The capacity of PB nanozymes to scavenge H_2_O_2_ was initially explored by utilizing the reaction of titanium sulfate with H_2_O_2_ to generate a yellow titanium peroxide-titanium complex with a characteristic peak at 415 nm. After PB nanozymes were added to the reaction system, the H_2_O_2_ was consumed and the absorbance at 415 nm decreased, and the clearance of H_2_O_2_ could be calculated. As seen in Fig. 4D, the H_2_O_2_ consumption by PB nanozymes was concentration-dependent, with a 73.3% H_2_O_2_ scavenging rate at a PBNPs concentration of 1000 ng/ml. CAT or CAT-mimics catalyze the decomposition of H_2_O_2_ to yield O_2_ and H_2_O, protecting the body from oxidative damage induced by H_2_O_2_. To further elucidate the CAT mimetic activity of PBNPs, the catabolism of H_2_O_2_ was assessed with disodium terephthalate (TPA-Na), and the non-fluorescent TPA-Na could transform H_2_O_2_ into a fluorescent indicator with a characteristic peak at 425 nm. In Fig. 4A, the fluorescence intensity at 425 nm decreased sharply in the presence of PBNPs, implying their competent CAT-like performance. Next, we evaluated the OH**·** depletion capacity of PBNPs by electron paramagnetic resonance (EPR) with 5,5-dimethyl-1-pyrroline N-oxide (DMPO) as a spin probe (Fig. 4E). The OH**·** is initially generated by the Fe^2+^/H_2_O_2_ reaction system with a distinct OH**·** signal in the EPR. And the OH**·** signal intensity dramatically declined in a concentration-dependent manner with the addition of PBNPs, attesting that the OH**·** signal was significantly attenuated after low-dose PBNPs disposal. POD is also an antioxidant enzyme that detoxifies H_2_O_2_ into water and can catalyze the oxidation of guaiacol to produce a tea-brown substance in the presence of H_2_O_2_, which has a maximum absorption at 470 nm. After adding different concentrations of PBNPs to the reaction system, the POD-like activity of PBNPs was measured by detecting the absorbance at 470 nm. And the highest POD-like activity of 45.1 U/ml was observed at 200 µg/ml of PBNPs (Fig. 4B). Finally, SOD is another classical antioxidant enzyme that turns O_2_^**·−**^ into H_2_O_2_ and O_2_, and an SOD assay kit was operated to quantify the SOD mimetic activity of PBNPs. The O_2_^**·−**^ was generated through the xanthine (X)/xanthine oxidase (XO) system, which convert the WST-1 into WST-1 formazan with an absorption peak at 450 nm (Fig. 4C). The SOD-like activity of PBNPs was also concentration-dependent, with the maximum SOD activity of 40.8% at 200 µg/ml of PBNPs. Taking DMPO as the spin probe, we further confirmed the ability of PBNPs to scavenge O_2_^**·−**^ by EPR. In the X/XO system, there appeared an intense characteristic signal peak of DMPO/O_2_^**·−**^. After the addition of PBNPs (10 µg/ml), the signal intensity dropped obviously, and when the concentration of PBNPs was further increased to 40 µg/ml, the signal intensity decreased steeply, indicating that PBNPs possessed superior scavenging ability for O_2_^**·−**^.

In addition, to assess the protective effect of PBNPs on cells against oxidative stress, 2′,7′-Dichlorofluorescin diacetate (DCFH-DA) was used to detect ROS levels in HaCaT cells. After cells were exposed to 750 µM H_2_O_2_, the intracellular ROS level in the positive control group raised to 73.13%, which was remarkably higher than that in the negative control group, and intracellular ROS levels were considerably lower in the pretreatment group with different concentrations of PBNPs compared to the positive control group. Importantly, the intracellular ROS level in the pretreatment group with 200 µg/ml of PBNPs was approached to that in the negative control group, signifying that 200 µg/ml of PBNPs could effectively avoid H_2_O_2_-induced elevation of intracellular ROS levels and maintain intracellular ROS at a normal level. Therefore, PBNPs with prominent ability to scavenge ROS are auspicious to reshape the harsh ROS microenvironment and serve as a promising antioxidant in deep second-degree burn wounds.

### Algs Hydrogels regulate the polarization of macrophages

Macrophages perform several critical functions throughout the wound healing process, recognizing and removing pathogens, cellular debris, and phagocytosing apoptotic neutrophils in the early stages, and forwarding angiogenesis, collagen deposition, and epithelial cell migration in the later phases [24–26]. Unfortunately, the phenotypic conversion of pro-inflammatory M1 macrophages into a pro-resolution state tends to be dysregulated in deep second-degree burn wounds, accounting for a poor regenerative environment [28]. Recently, it has been demonstrated that sulfated GAGs can induce the polarization of macrophages from M1 to M2 [53]. Sulfated GAGs with highly negatively charged groups can modulate the distribution of some heparin-binding proteins within the ECM [17–19], which, in turn, eases the terrible inflammatory environment around macrophages. Furthermore, some unique sulfated GAGs with pentasaccharide sequence, such as sulfated chitosan, directly interact with macrophage surface receptors, thus activating pathways associated with macrophage polarization [53, 54]. Therefore, we speculate that Algs sharing similar structure with sulfated chitosans, can also trigger the macrophages transition from M1 to M2.

**Fig. 5:**
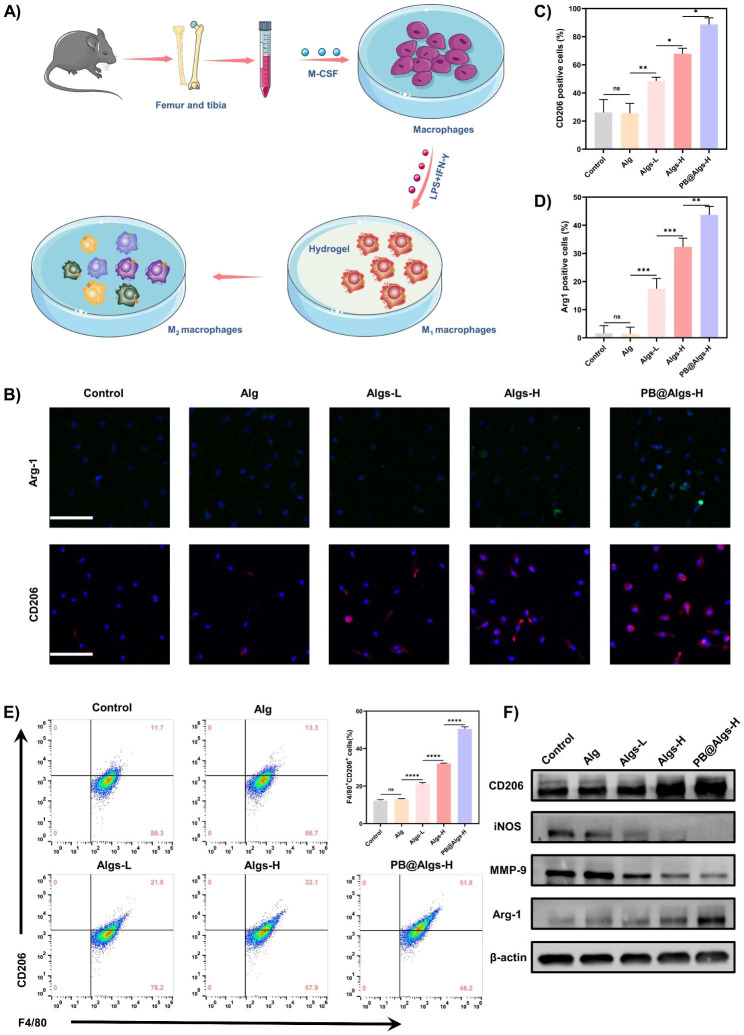
Macrophage polarization in response to Algs hydrogels in vitro. (**A**) Illustration of in vitro bone marrow-derived macrophage (BMDMs) stimulation assays. (**B**) Representative immunofluorescence staining images of CD206 and Arg-1 (M2 macrophages marker) of BMDMs. Scale bar: 100 μm. **C, D**) Statistical data of the percentage of Arg-1^+^ and CD206^+^ macrophages. **E**) Flow cytometry analysis indicating the proportion of M2 macrophages (F4/80^+^CD206^+^). **F**) Protein expression level of CD206, iNOS, MMP-9, and Arg-1 in macrophages determined by western blot. (n = 3; mean ± s.d.; ns, not statistically significant; *p < 0.05, **p < 0.01, ***p < 0.001, ****p < 0.0001)

To verify this hypothesis, F4/80 and CD11b double-positive macrophages of bone marrow origin were first extracted, purified, and characterized by flow cytometry (Fig. 5A). Next, lipopolysaccharide (LPS) and interferon-γ (IFN-γ) were employed to stimulate bone marrow-derived macrophages to polarize toward M1 (Figure S3A). After completing these pre-processes, we analyzed the effects of hydrogels on M1 macrophages by immunofluorescence. M1 macrophages were induced to substantially express CD206 and Arg-1 associated with the M2 macrophages in the cell membrane and cytoplasm after co-incubation with Algs-L, Algs-H, and PB@Algs-H hydrogels (Fig. 5B-D). Among them, the PB@Algs-H group obtained the maximum expression of CD206 and Arg-1, while the fluorescence intensity of the control and Alg groups were weak and there was no apparent difference between them. We quantified the proportion of M2 macrophages (F4/80^+^, CD206^+^) after stimulation with hydrogels by flow cytometry in parallel. The PB@Algs-H group acquired the largest proportion of M2 macrophages, in agreement with the immunofluorescence results (Fig. 5E). Then, we quantitatively analyzed the relative mRNA expression of M1 macrophage marker genes (iNos, TNF-α, IL-6, and Il-1β) and M2 macrophages marker genes (Arg1, Il-10, and VEGF) by RT-qPCR (Figure S3F and G). The results of RT-qPCR remained consistent with previous findings. Finally, the expression of M1 macrophages markers (iNOS and MMP-9) and M2 macrophages markers (CD206 and Arg-1) were detected by western blot. The relative expression of M1 macrophages markers was significantly lower in the Algs-L, Algs-H, and PB@Algs-H groups compared to the control and Alg groups, while the relative expression of M2 macrophage-related markers was markedly higher (Fig. 5F and Figure S3B-E), which was coherent with the results of immunofluorescence and flow cytometry. Clearly, Alg failed to induce the phenotypic conversion of M1 macrophages, whereas Alg owned such a property after being sulfated, authenticating our conjecture that the introduction of the sulfate group granted it with this function. In fact, the above results also uncovered that the PB@Algs-H group possessed stronger potential to facilitate the polarization of M1 macrophages compared to the Algs-H group. This may be related to the fact that PB nanozymes in PB@Algs-H hydrogels scavenged ROS in the culture environment and inhibited the stimulation on macrophages from ROS, which has been reported in the literature. The reduction of M1 macrophage-derived inflammatory mediators, such as tumor necrosis factor-α (TNF-α), inducible nitric oxide synthase (iNOS), and proteases (e.g., MMP-9) will modulate the wound microenvironment, attenuate tissue damage, and improve growth signals. In contrast, the ornithine from M2 macrophage-associated Arg-1 can promote cell proliferation and VEGF secretion, which may bolster neovascularization, thereby raising the potential for tissue regeneration and recovery.

### Effects of the hydrogels on Deep Second-Degree burn wounds in vivo

**Fig. 6:**
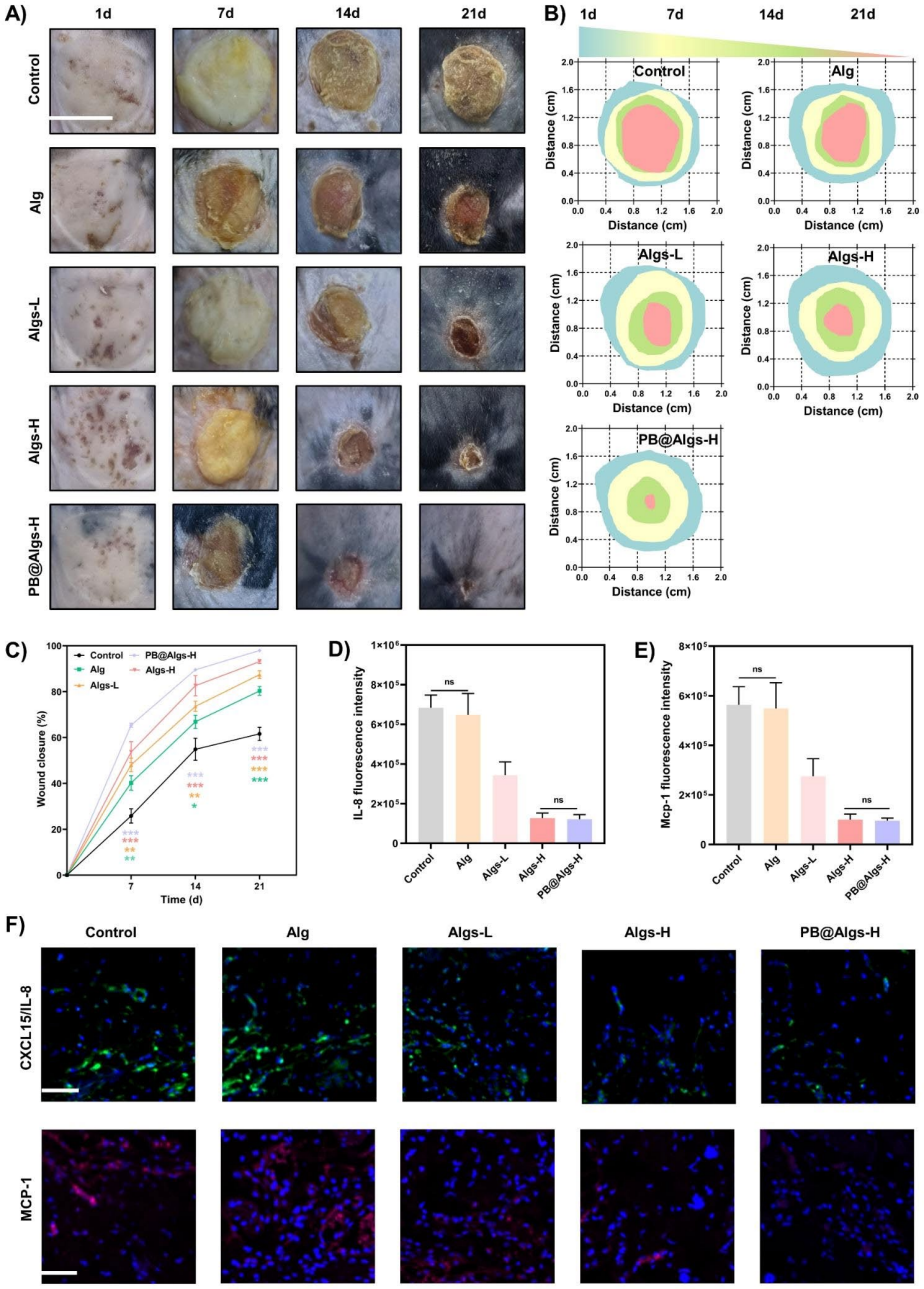
Effects of the hydrogels on promoting deep second-degree burn wound healing in mice. (**A**) Representative images of wound healing process in deep second-degree burn wound mice. Scale bar: 1 cm. (**B**) Schematic diagram of the wound healing treated with hydrogels during 21 days. (**C**) Quantitative data of relative wound closure at different time points. **D, E**) Statistical data of IL-8/CXCL15 and MCP-1 fluorescence intensity. **F**) Representative IL-8/CXCL15 and MCP-1 immunofluorescence staining images on day 2 after treatment with hydrogels. Scale bar: 100 μm. (n = 3; mean ± s.d.; ns, not statistically significant; *p < 0.05, **p < 0.01, ***p < 0.001)

In vitro studies have proven that Algs hydrogels possessed powerful anti-inflammatory capacity. Therefore, a deep second-degree burn model in mice was made to evaluate the therapeutic effects of hydrogels. To acquire accurate burn area and depth, an electric scalding instrument with controlled temperature and pressure was utilized for modeling. Compared with the normal skin tissue of mice, the whole epidermis and deep dermis of the experimental group were devastated, and only the deep skin attachment remained, but the subcutaneous fat layer and muscle tissue were structurally intact and undamaged. Importantly, the burn degree remained consistent across experimental groups, minimizing its influences on the final results (Figure S4A). Next, deep second-degree burn wounds in mice were treated with saline dressing (control group), Alg, Algs-L, Algs-H, and PB@Algs-H hydrogels and assessed by observation of healing rate and tissue collection on days 1, 7, 14, and 21. Macroscopically, four hydrogel-treated groups (Alg, Algs-L, Algs-H, and PB@Algs-H) healed faster in comparison with the control group, evidencing the therapeutic effect of hydrogels. Among them, the PB@Algs-H group attained the highest healing rate, with 65%, 90%, and 98% healing ratio on days 7, 14, and 21, respectively, and only 26%, 55%, and 62% wound closure in the control group (Fig. 6B and C). The control group received virtually invalid treatment, and the deplorable wound inflammation need to be resolved on its own, which is a hard and long-term process. Interestingly, in vitro studies have disclosed that Alg is almost extraneous to anti-inflammation, but the Alg group had a faster healing rate than the control group. The result may probably attribute to the fact that the hydrogels vigorously absorbed the exudate, induced autolysis of necrotic tissue, and accelerated wound bed autodebridement, which is noteworthy to alleviate wound inflammation. The persistence of necrotic tissue on the wound bed will recruit a constant flow of inflammatory cells, aggravating the wound inflammation. Consequently, this may also be one of the reasons for the wide application of Alg hydrogels in clinical burn wounds.

**Fig. 7:**
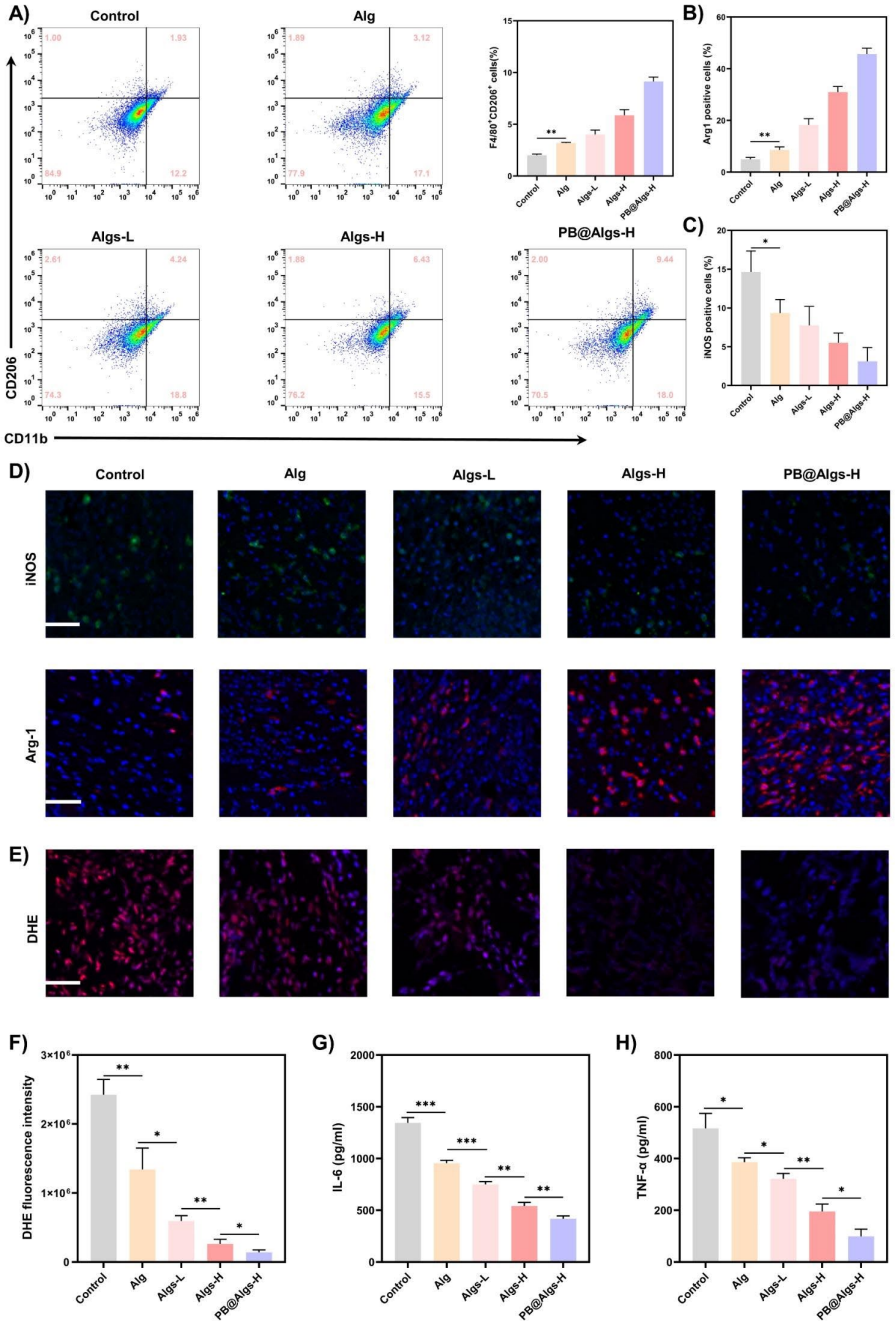
Anti-inflammatory effects of hydrogels on deep second-degree burn wounds. **A**) Flow cytometry analysis indicating the proportion of M2 macrophages (CD11b^+^CD206^+^) on day 7. **B, C**) Statistical data of the percentage of Arg-1^+^ and iNOS^+^ macrophages. **D**) Representative immunofluorescence staining images of iNOS and Arg-1 on day 7. Scale bar: 100 μm. **E**) DHE staining images indicating ROS levels in wound tissue on day 7. Scale bar: 100 μm. **G, H**) Concentrations of TNF-α and IL-6 in wound tissue measured by ELISA on day 4. (n = 3; mean ± s.d.; *p < 0.05, **p < 0.01, ***p < 0.001)

Wound healing is an orchestrated cascade with four overlapped but distinct phases: hemostasis, inflammation, proliferation, and remodeling [55]. Any endogenous and exogenous adverse factors can disrupt the physiological healing process, of which the inflammatory phase is the most susceptible to disturbance [56, 57]. The deep second-degree burn wounds are characterized by abundant inflammatory cells infiltration and a high level of pro-inflammatory cytokines in the early stages due to severe tissue damage. Unfortunately, the awful inflammatory environment precludes the transition from the inflammatory to the proliferative phase of the wound healing [4–6]. In this context, we evaluated the impact of hydrogels on the inflammatory phase of wound repair. We first analyzed the IL-8/CXCL15 and MCP-1 adsorption by hydrogels using immunofluorescence on day 2 (IL-8 is not present in mice and CXCL15 is its corresponding mouse homologue). There was no obvious difference in the presence of these two chemokines between Alg and the control group, both of which shared a wide prevalence. Whereas the fluorescence intensity of these two critical inflammatory mediators was drastically declined in the Algs-H and PB@Algs-H groups compared to the Alg and control groups, and there was no visible difference between these two groups, which was consistent with in vitro results (Fig. 6D-F). The persistence of numerous IL-8/CXCL15 and MCP-1 in the wound will attract more inflammatory cells such as neutrophils and monocytes, initiating a vicious cycle of wound inflammation. Next, on day 4, we measured the expression of two other key inflammatory factors, TNF-α and IL-6, by ELISA. As a secondary effect of the repressed accumulation of immune cells in the wound, the TNF-α and IL-6 expression in Algs-contained groups was considerably decreased relative to the control group and Alg groups, especially in the PB@Algs-H group, in which PB nanozymes removed substantial ROS besides the chemokine sequestering effect of Algs-H (Fig. 7G and H). On top of that, we found that there was a downregulation of TNF-α and IL-6 expression in the Alg group over the control group, which is aligned with the observed outcomes of wound healing. While Alg appears to be irrelevant to the resolution of inflammation, their role in accelerating the process of autolysis and inducing autolytic wound cleansing is reconfirmed. Then, the in vivo antioxidant capacity of PB nanozymes was validated by employing a ROS-sensitive dihydroethidium (DHE) probe to detect ROS levels in wound tissue. In comparison with the control group, the DHE fluorescence intensity was evidently depressed in the hydrogel groups, among which the PB@Algs-H group reflected the weakest fluorescence intensity, thoroughly testifying to the antioxidant activity of PB nanozymes. although Alg or Algs itself lacked evident antioxidant activity, the DHE fluorescence intensity of the Alg, Algs-L, and Algs-H groups weakened sequentially. The inflammatory microenvironment in deep second-degree burn wounds is extremely complex, which varies as Alg or Algs hydrogels progressively exercise their unique therapeutic effects on the wound, thus affecting the production and quenching of ROS. This may account for the popularity of extensive functional hydrogels. The inflammatory phase of deep second-degree burn wounds is noticeably prolonged, which is also relevant to the failure of phenotype switch of macrophages timely. In vitro studies have already evidenced the potential of Algs to induce the polarization of M1 macrophages toward M2-like macrophages. The effect of hydrogels on M1 macrophages in deep second-degree burn wounds was further investigated. On day 7, the junction of the inflammatory and proliferative phases, wound tissue was collected and prepared as single cell suspensions for flow cytometry to quantify the proportion of M2 macrophages. The proportion of M2 macrophages (CD11b^+^, CD206^+^) was distinctly higher in the Algs-L, Algs-H, and PB@Algs-H groups than that in the untreated group, which remained consistent with the in vitro results (Fig. 7A and S3H). Interestingly, we found that the Alg group owned a higher percentage of M2 macrophages in comparison to the control group, which was not corresponding with the in vitro results. This may still be pertinent to the therapeutic effects of Alg hydrogels we discussed previously, and the alteration of the inflammatory wound environment brought a phenotype shift of macrophages. Next, the impacts of hydrogels on M1 macrophages were further evaluated by immunofluorescence staining on M1 macrophages (iNOS) and M2 macrophages (Arg-1). The results confirmed the trends observed in thus far, with the PB@Algs-H group achieving the highest M2/M1 macrophage ratio of all groups (Fig. 7B-D). On day 7, the density of M1 macrophages was lower in the hydrogel-treated group, while the density of M2 macrophages was comparatively higher than that in the control group, indicating that the wound healing process was gradually progressing into the proliferative phase. In contrast, plenty of iNOS^+^ macrophages were still visible in the control group, denoting that the inflammatory phase was still ongoing. With the timely phenotype transformation of M1 macrophages, the multifunctional hydrogels further curbed the inflammatory response in wound healing, which, in turn, boosted angiogenesis and tissue remodeling. Accordingly, the PB@Algs-H hydrogels put remarkable anti-inflammatory effects on deep second-degree burn wounds and accelerated the transition from the inflammatory phase to the proliferative stage, subsequently encouraging tissue regeneration.

**Fig. 8:**
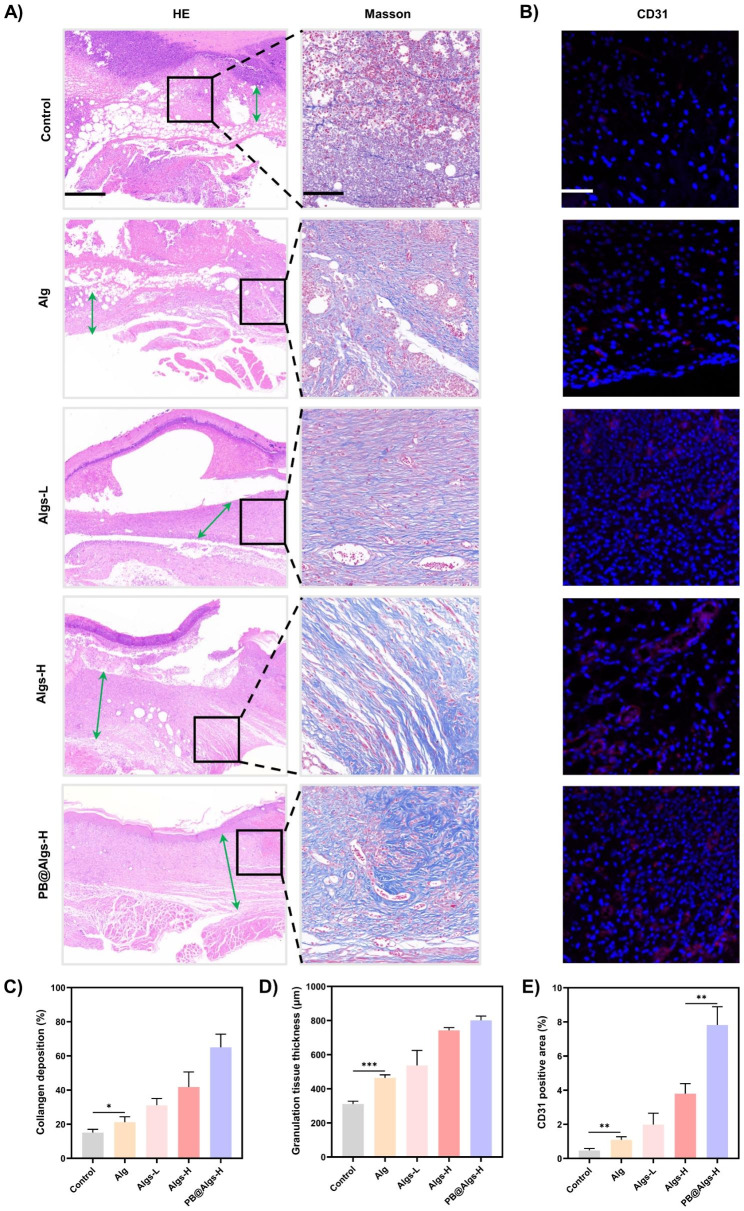
Effects of anti-inflammatory hydrogels in the proliferative and remodeling phases of wound healing. (**A**) HE staining of the wound tissue reflected the thickness granulation tissue (green arrows) on day 21. Scale bar: 500 μm. Masson’s trichrome staining of the wound tissue reflected collagen deposition area on day 21. Scale bar: 100 μm. (**B**) Representative immunofluorescence staining images of CD31 reflected neovascularization. Scale bar: 100 μm. (**C**) Statistical data of collagen deposition area. (**D**) Statistical data of granulation tissue thickness. (**E**) Statistical data of CD31 positive area in the wound tissue. (n = 3; mean ± s.d.; *p < 0.05, **p < 0.01, ***p < 0.001)

Next, we assessed the influences of hydrogels on wound tissue regeneration and remodeling. During the proliferative phase, neovascularization is critical to ensure adequate nutrient and oxygen delivery to the wound tissue to support fibroblast proliferation, collagen synthesis, and re-epithelialization [58]. Immunostaining for platelet endothelial cell adhesion molecule-1 (CD31) was implemented on day 14 to inspect the presence of neovascularization in the granulation tissue. The CD31 expression was greater in hydrogel groups than that in the control group, with the highest expression level in PB@Algs-H group, which is line with with the healing results observed under macroscopy (Fig. 8B and E). In the remodeling phase, proper collagen deposition and remodeling is necessary to enhance the tensile strength of the tissue and support wound healing. The formation of new collagen was observed by Masson’s trichrome staining, and the thickness of granulation tissue in H&E staining was examined synchronously. On day 21, profuse residual dry scabs could still be observable in the control group, implying a delayed healing of deep second-degree wounds (Fig. 8A). On the contrary, the hydrogel-treated wounds exhibited a thicker granulation layer and more collagen deposition (Fig. 8C and D). Among them, the PB@Algs-H group displayed the greatest thickness of granulation tissue and the widest range of collagen deposition, disclosing that the damaged tissues in the PB@Algs-H group were close to recovery and maturation and approaching normal skin. The favorable performance of the proliferative and remodeling phases in hydrogel groups was attributed to the fact that Algs and PB nanozymes greatly restricted the headway of disproportionate inflammation in the early stages and expedited the progression of the wound healing from the inflammatory to the proliferative phase.

**Fig. 9:**
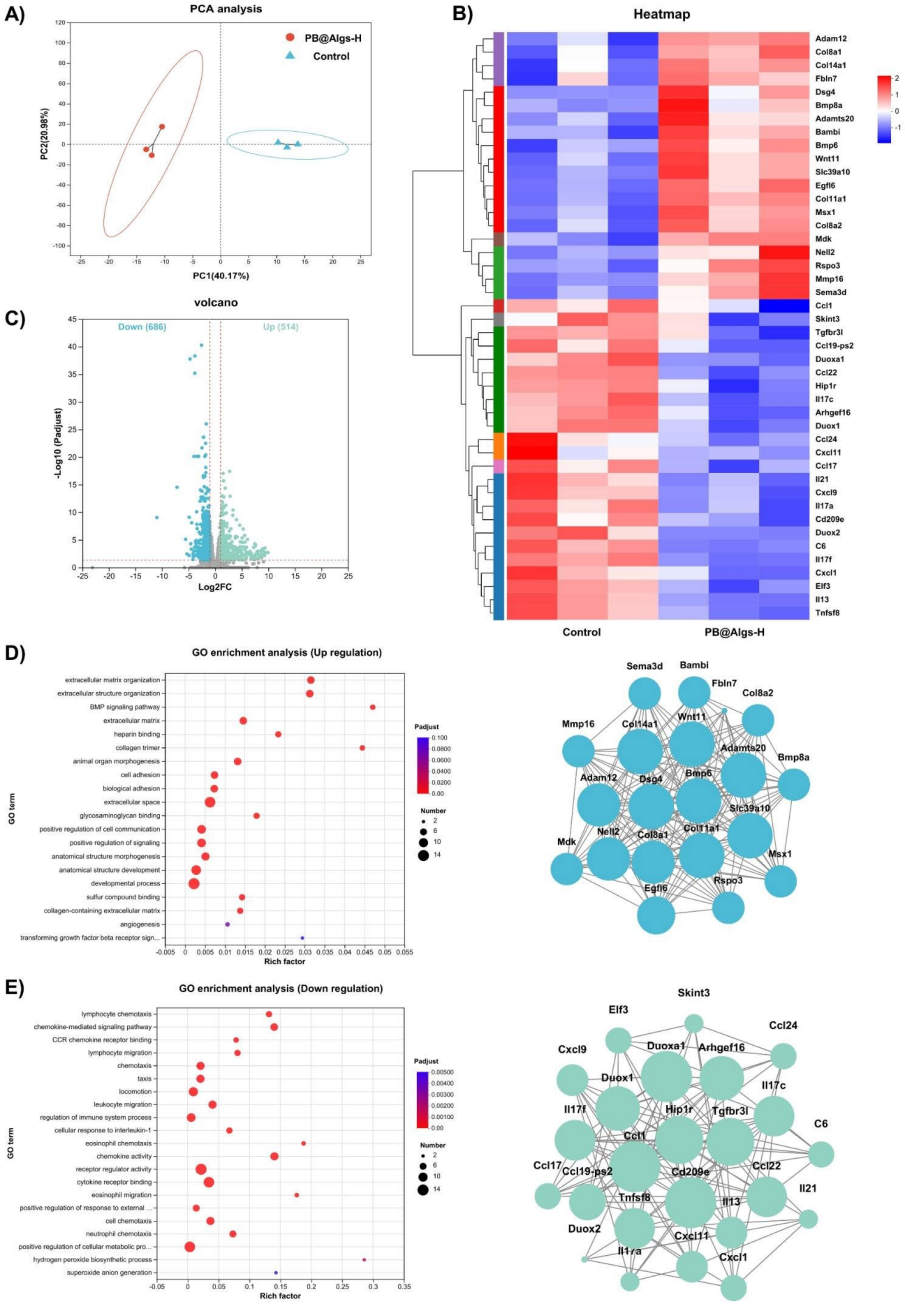
Comprehensive assessments of the wound microenvironment after treatment with PB@Algs-H hydrogels using RNA sequencing. (**A**) PCA based on DEGs in the wound tissue between the control and PB@Algs-H groups. (**B**) Heat map of upregulated and downregulated genes in the deep second-degree burn wound microenvironment after treatment with PB@Algs-H hydrogels (fold change ≥ 4 and P < 0.05). (**C**) Volcano plots showing the upregulated and downregulated genes in response to PB@Algs-H hydrogels treatment. (**D**) GO enrichment analysis of the upregulated genes. (**E**) GO enrichment analysis of the downregulated genes

To comprehensively evaluate the wound microenvironment after treatment with PB@Algs-H hydrogels, RNA sequencing was performed on wound tissue on day 7 according to the in vivo experiment results, and the untreated deep second-degree burn wound tissue was taken as a control. The unguided principal component analysis (PCA) showed significant differences in transcriptomic profiles between the control and PB@Algs-H groups (Fig. 9A). According to the empirical Bayes method, 1200 significantly differentially expressed genes (DEGs) were identified after administration of PB@Algs-H hydrogels, of which 514 were upregulated and 686 were downregulated, as shown in the volcano plot (Fig. 9C). Then, DEG_S_-based enrichment analysis was conducted on the up- and down-regulated gene sets. Hierarchical clustering analysis screened and separated for differences in gene expression between the control and PB@Algs-H groups of mouse wound tissue (Fig. 9B). Particularly, the expression of genes involved in wound inflammation (*Ccl24, Ccl1, Il17a, Cxcl1, Cxcl9, Ccl22, Ccl17, and Cxcl11*) was significantly downregulated after PB@Algs-H treatment, as were genes related to ROS production (*Duox1, Duox2, and Duoxa1*). Nevertheless, genes associated with tissue regeneration and proliferation (*Egfl6, Col14a1, Adamts20, Col11a1, Mmp16, Col8a2, and Col8a1*), and genes involved in heparin protein binding (*Rspo3, Nell2, Mdk, Fbln7, and Col11a1*) were significantly upregulated. Gene ontology (GO) analysis uncovered that the upregulated genes were mainly focused on extracellular matrix synthesis and heparin protein binding (Fig. 9D), while the downregulated genes were mostly associated with inflammatory cell migration and oxidative stress (Fig. 9E). The results revealed that the aggregation of inflammatory cells to the wound bed was promptly interrupted and the superfluous synthesis of ROS was restrained in treated deep second-degree wounds. And the immoderate inflammatory infiltration of the burn wounds was relieved, while exhibiting excellent regenerative potential and positive response to growth signals.

Next, up and down-regulation datasets were collected for Kyoto Encyclopedia of Genes and Genomes (KEGG) pathway enrichment analysis. KEGG pathway enrichment analysis revealed that PB@Algs-H hydrogels potently suppressed some inflammatory pathways such as chemokine signaling pathway and IL-17 signaling pathway (Figure S5A), which were correlated with progressive wound inflammation. Inhibition of these pathways confirmed the anti-inflammatory effects of PB@Algs-H hydrogels in deep second-degree wounds. Furthermore, The KEGG of up-regulation dataset was focused on TGF-β signaling pathway and Wnt signaling pathway (Figure S5B), which were associated with cell migration, proliferation, and ECM reconstruction. Dynamic crosstalk in the WNT and TGF-β signaling pathways might greatly advance the wound healing progression. The mitigation of hyperinflammation and activation of growth signaling might deliver an improved healing outcome. To verify the RNAseq results, we quantitatively analyzed key genes in these pathways described above, and the RNA relative expression levels were consistent with KEGG results (Figure S5C and D).

## Discussion

Currently, the non-surgical treatment of deep second-degree burn wounds mainly includes infection control, wound debridement, and wound dressing coverage. The most commonly applied wound dressings are hydrogels, which absorb the bulk of wound exudate but most often lack active elements that can regulate the wound inflammatory microenvironment [59, 60], while the predicament in the management of refractory wounds lies in the unrestrained hyperinflammation. Though some bioactive substances, such as immunosuppressants and stem cells, have been widely studied, they are challenged by their susceptibility to inactivation, low bioavailability, limited modulatory effects on wound inflammation. Recently, several GAGs-based hydrogels have been exploited for inflammatory modulation of wounds, such as heparin, sulfated chitosan, etc. [13, 14, 53]. Compared with other means of inflammation regulation, the regulation based on biomolecular interactions is not only sustainable and effective, but also affordable, which will vastly benefit future clinical practice. Nevertheless, it is also essential to recognize that all of them are currently confronted with complex preparation processes and numerous side effects. Therefore, there is an urgent requirement for the design of wound dressings that can safely, effectively, and stably modulate the wound inflammatory microenvironment. On the premise of outstanding biocompatibility, Alg and PB, which have been widely applied clinically, were selected as substrates. Algs were synthesized by a one-step method and readily prepared PB antioxidant nanozymes with high efficiency were loaded in the hydrogel and obtained steady release from the hydrogel platform. Additionally, the Algs hydrogels were tailored with a soft property and ECM-mimetic structure to achieve mutual acclimatization of hydrogels with fragile deep second-degree burn wounds. To address the previous issue on few targets for inflammation regulation, we adopt a multipronged approach to modulate the wound inflammation from three dimensions: chemokines, M1 macrophages, and ROS, and thus the deep second-degree burn wounds were rescued from desperate inflammatory impairment and harvested a rapid transition from the inflammatory to the proliferative phase. Finally, the clinical translation of therapeutic PB@Algs-H composite hydrogel dressings will be facilitated by their robust anti-inflammatory properties in the complex refractory wounds and absence of any bioactive ingredients, such as VEGF. Beyond cutaneous wound healing, the novel anti-inflammatory technologies based on PB@Algs-H hydrogels are promising to blaze a trail for the therapy of other diseases associated with excessive inflammation, such as ulcerative colitis, arthritis, sepsis, etc.

In fact, the inflammatory milieu in wounds is extremely complex. Low levels of inflammation in the early phase are susceptible to infection, while hyperinflammation infiltration in the later stages will disrupt normal healing events. Therefore, monitoring the level of wound inflammation is essential during the treatment process. The combination of anti-inflammatory Algs with wound microenvironment monitoring will be more promising. In the future, anti-inflammatory hydrogel dressings may allow for precise regulation during healing processes, with real-time monitoring of wound inflammatory microenvironment by imaging devices and wearable sensors. Such progressive advancements in precise treatment of various wounds will raise patient cure rates, alleviate pain, and lower costs.

## Conclusion

Deep second-degree burn wounds with unique inflammatory processes in pathophysiology are different from other wounds, such as abrasions and lacerations. Radical treatments, such as surgery, are commonly painful and financially burdensome for patients, while conservative treatments frequently face failure. For deep second-degree burn wounds, the goals of management and treatment include mitigating pain, minimizing scarring, and advancing rapid healing, with the ultimate goal of restoring full function and visual aesthetics to the injured region. Notably, our hydrogel platform may solve this challenge. The superior biocompatibility of Algs and PBNPs paves the way for clinical application, and the soft nature and the porous ECM-like structure of Algs hydrogels allow for better accommodation to the evolution of deep second-degree burn wounds. Meanwhile, a dual anti-inflammatory strategy consisting of Algs and PBNPs realizes the goal of regulating the uncontrolled wound inflammation, thus ensuring a rapid transition from the inflammatory to the proliferative phase of wound healing and ultimately reaping favorable healing outcomes. This further confirms that the combination of modulating the immune response and enhancing the mutual adaptation of hydrogels with wounds is an effective and promising pathway for the treatment of delayed skin wound healing in deep second-degree burn conditions.

### Electronic supplementary material

Below is the link to the electronic supplementary material.

Supplementary Material 1

## Data Availability

All data generated or analyzed during this study are included in the article and additional file.
