# Supplementary material for: Tunable Sulfated Alginate-based Hydrogel Platform with enhanced anti-inflammatory and antioxidant capacity for promoting burn wound repair
Source: J Nanobiotechnology. 2023 Oct 24;21:387. doi: 10.1186/s12951-023-02144-2 (PMC10594798; doi:10.1186/s12951-023-02144-2)
Supplement: Supplementary file 1 — Supplementary Material 1 [file 12951_2023_2144_MOESM1_ESM.docx]

**Supporting Information**

**Tunable Sulfated Alginate-based Hydrogel Platform with Enhanced Anti-inflammatory and Antioxidant Capacity for Rescuing Burn Wound Repair**


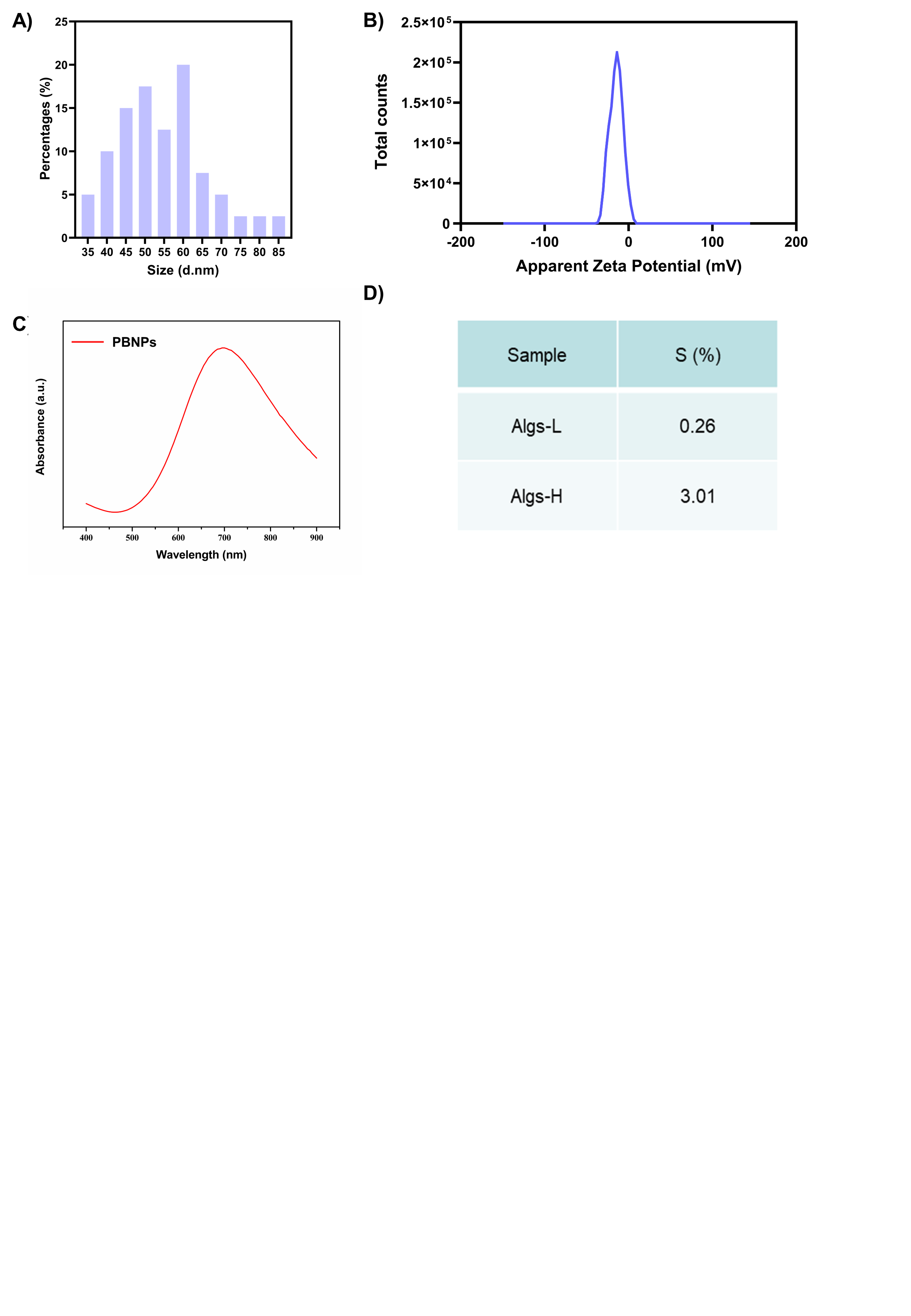


**Figure S1.** A) Particle size distribution of PBNPs. B) Zeta potential of PBNPs. C) UV–vis–NIR absorbance curve of PBNPs. D) Sulfur content in the sulfated alginate samples determined by elemental analysis with ICP-OES.


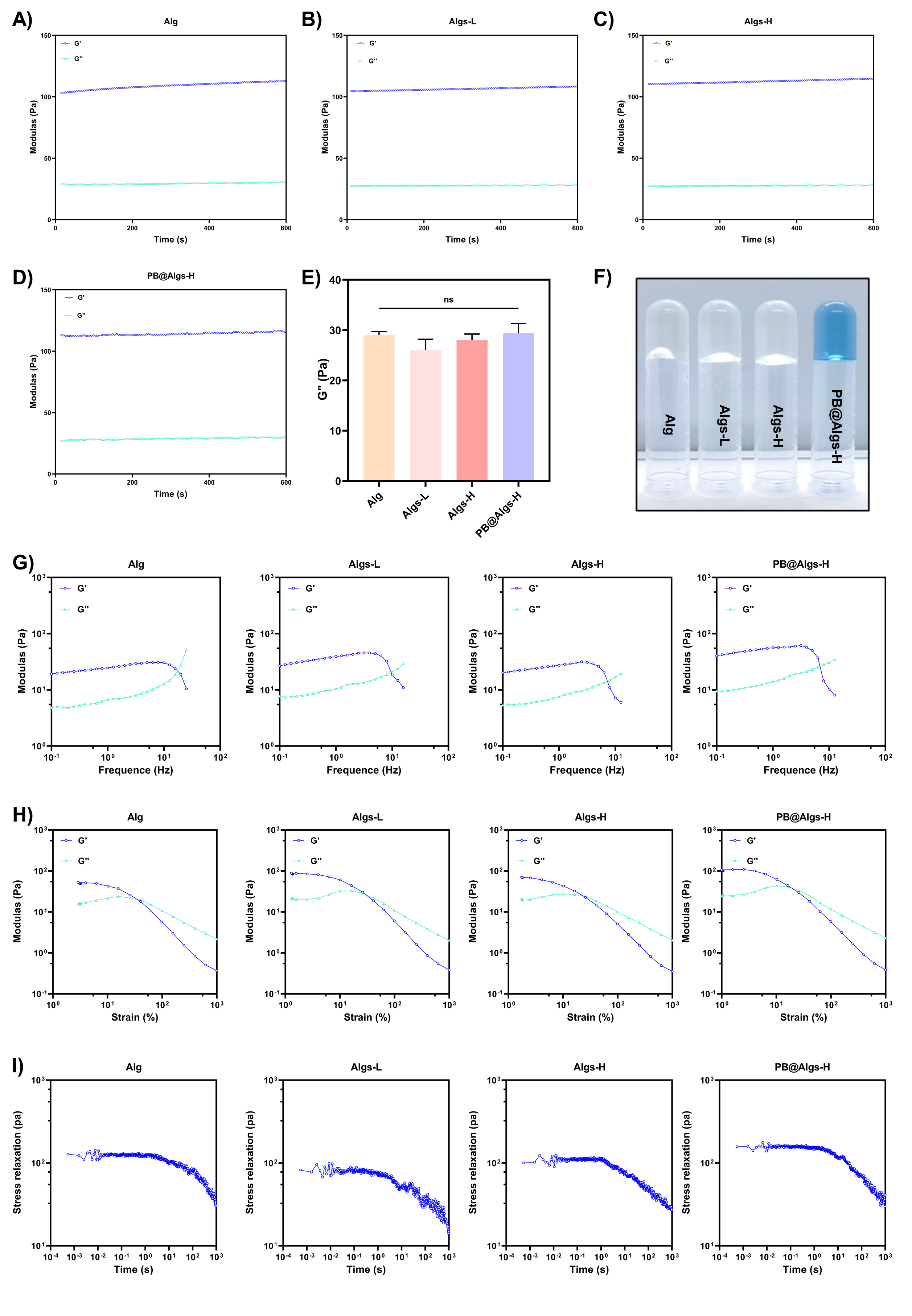


**Figure S2.** A, B, C, D) Storage modulus(G') and loss modulus (G'') with time of hydrogels. E) Loss modulus (G'') of hydrogels. F) Photographs of hydrogels at room temperature. G, H, I) Frequency sweep, strain sweep, and stress relaxation of hydrogels.


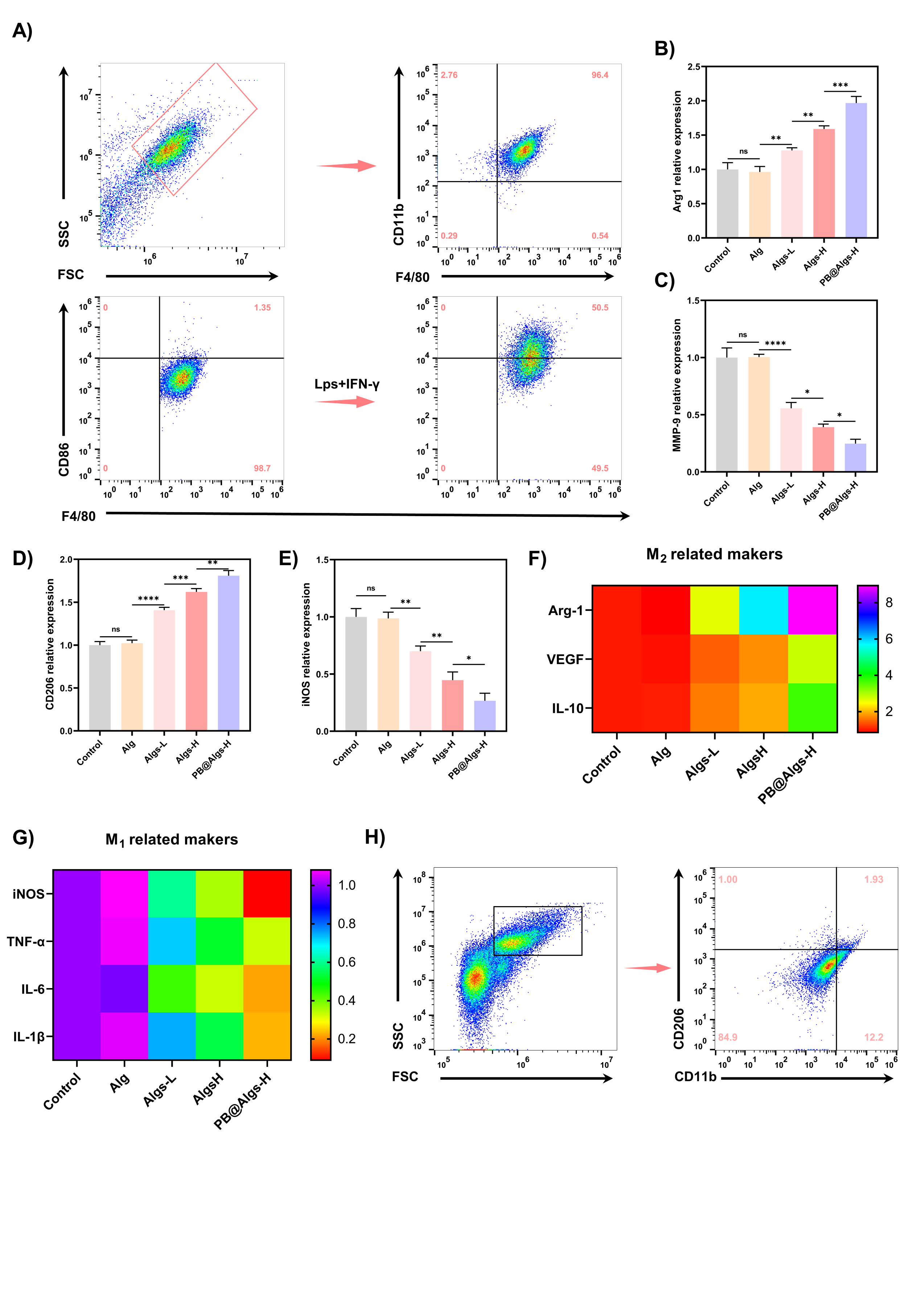


**Figure S3.** A) Flow cytometry analysis indicating the expression levels of F4/80 and CD11b in BMDMs and the proportion of M1 macrophages (F4/80^+^CD86^+^) after stimulation with LPS and IFN-γ. B, C, D, E) The relative expression levels of iNOS, MMP-9, CD206, and Arg-1 in each group. F, G) Heat map for expression analysis (RT-qPCR) of M1 and M2 macrophages marker genes. H) the gating strategy for flow cytometry in vivo. (n = 3; mean ± s.d.; ns, not statistically significant; *p < 0.05, **p < 0.01, ***p < 0.001, ****p < 0.0001)


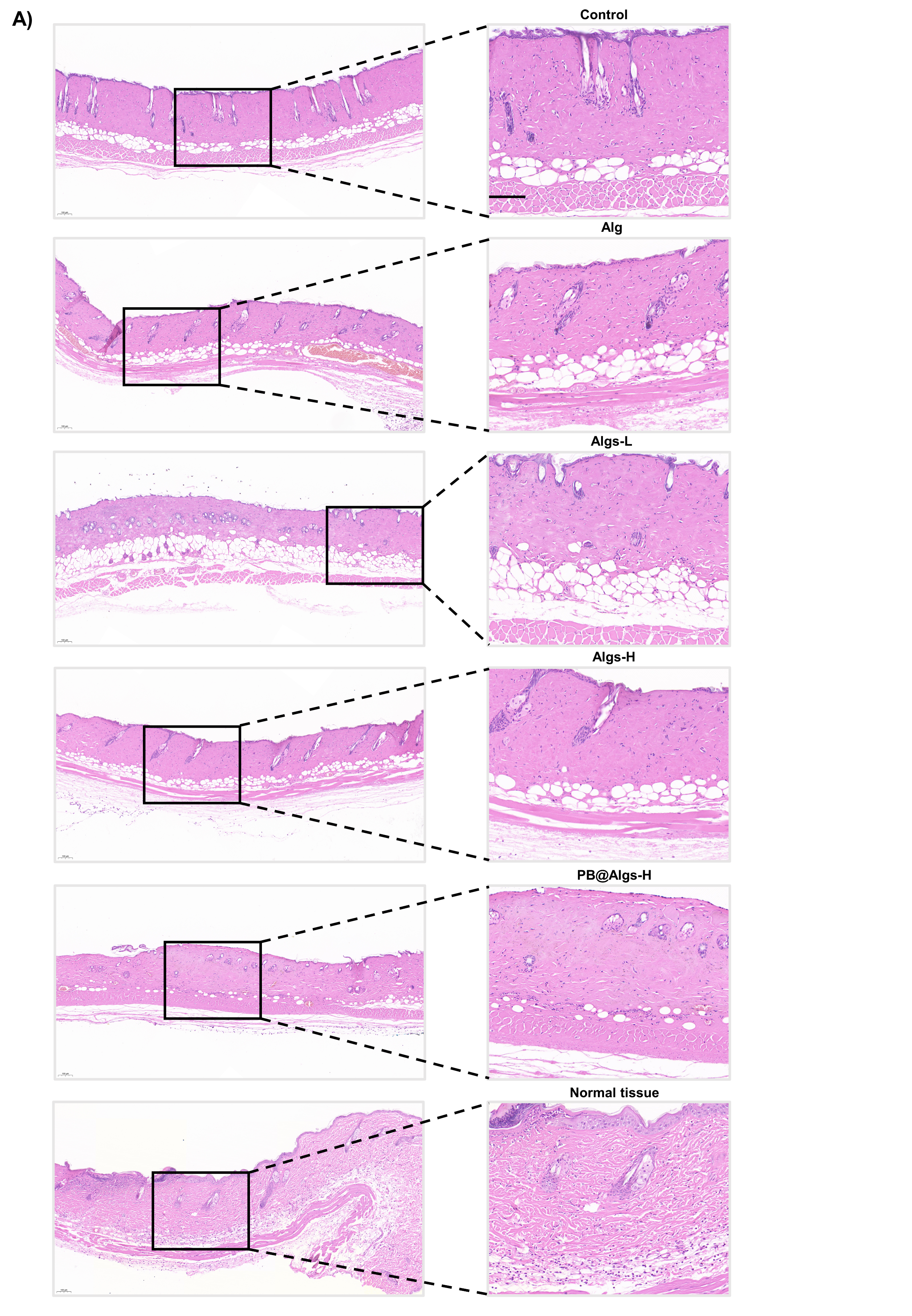


**Figure S4.** H&E staining reflected the burn degree on wound tissue. Scale bar: 100 µm.


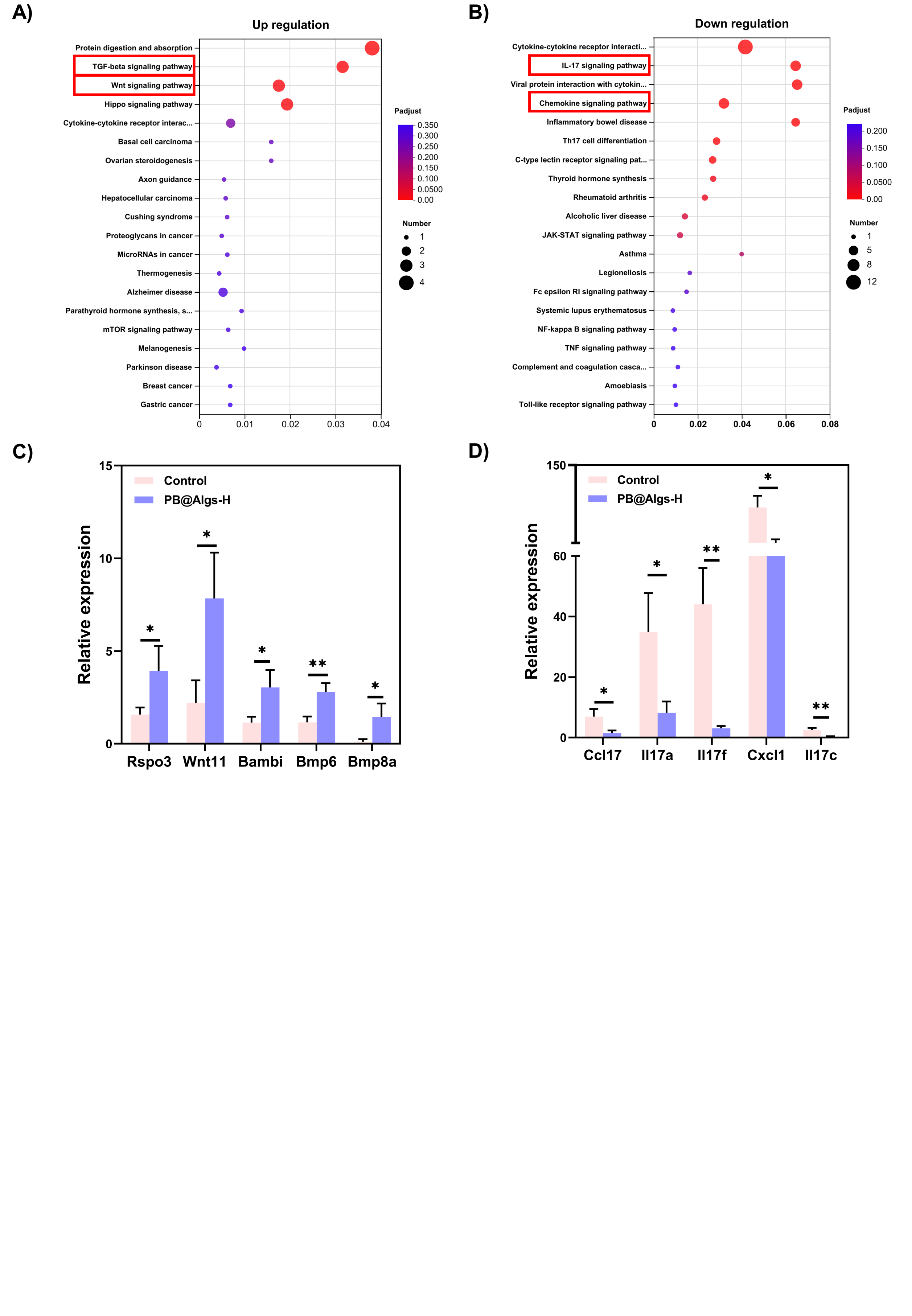


**Figure S5.** A, B) Up and down-regulation datasets in KEGG pathway enrichment analysis. C, D) RNA relative expression levels of key genes in up and down-regulated signaling pathways.
